# Use of Wet Milling Combined with Temperature Cycling to Minimize Crystal Agglomeration in a Sequential Antisolvent–Cooling Crystallization

**DOI:** 10.1021/acs.cgd.1c01510

**Published:** 2022-07-19

**Authors:** Zhuang Sun, Justin L. Quon, Charles D. Papageorgiou, Brahim Benyahia, Chris D. Rielly

**Affiliations:** † Future Continuous Manufacturing and Advanced Crystallization (CMAC), Research Hub at the Department of Chemical Engineering, Loughborough University, Loughborough, Leicestershire LE11 3TU, United Kingdom; ‡ Process Chemistry and Development, Takeda Pharmaceuticals International Company, 40 Landsdowne Street, Cambridge, Massachusetts 02139, United States

## Abstract

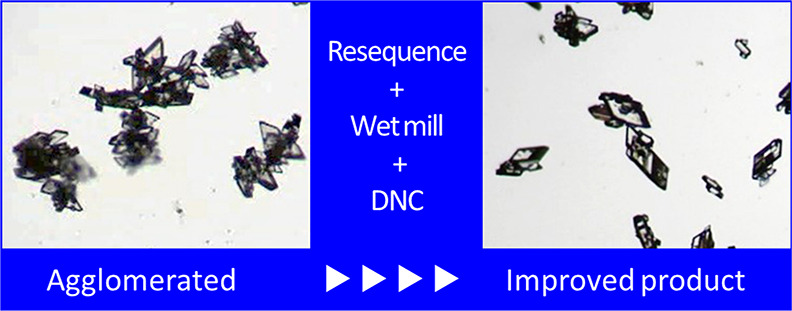

The objective of the research was to improve the process design of a combined antisolvent–cooling crystallization to reduce the degree of agglomeration of a real active pharmaceutical ingredient product, which was manufactured using a crystallization stage employing a methanol/water solvent system. Knowledge was gained from the use of process analytical technology (PAT) tools to monitor the process variables, allowing particle size, degree of agglomeration, solute concentration, and supersaturation to be tracked throughout the process. Based on knowledge of the solubility behavior and interpretation of the PAT histories, changes were made to the sequences of antisolvent addition and cooling within the crystallization process to reduce agglomeration in the final product. Different seed loadings and seeding addition points were also investigated to maintain operation within lower supersaturation regions of the phase diagram to limit agglomeration and avoid an undesired polymorphic transformation to an unstable form. The improved sequences of operations and seeding conditions did not provide sufficient improvement in the product quality and so were augmented by applying wet milling for further deagglomeration followed by temperature cycling to remove fine particles generated during milling. Open-loop heating and cooling cycles produced some limited improvements, whereas closed-loop direct nucleation control methods using FBRM as a feedback sensor for particle counts per second were much more successful at producing high-quality crystals of the desired polymorphic form. The work shows that understanding the trajectory of the process through the phase diagram to follow appropriate supersaturation profiles gives improved control of the various kinetic mechanisms and can be used to improve the quality of the final product.

## Abstract 2

Process design of a combined antisolvent−cooling crystallization of a real active pharmaceutical ingredient was improved using knowledge gained from PAT tools to track changes in particle size, degree of agglomeration, solute mass, and supersaturation. Resequencing operations using wet-milling and feedback-enabled direct nucleation control gave larger crystals of the desired polymorph with reduced agglomeration.

## 1 Introduction

Crystallization is a unit operation widely applied in the pharmaceutical, fine chemical, and food industries for the purification and control of product characteristics, such as crystal size and shape.^1^ Generally, the workflow for designing a crystallization process consists of five steps: (1) assessment of product chemical and physical information, (2) solvent screening and selection, (3) selection and calibration of process analytical technology (PAT) tools, (4) system and process understanding, and (5) design and proof of concept demonstration of the crystallization.^2^ In this work, the design of a batch sequential antisolvent and cooling crystallization of a Takeda API (compound X) was investigated experimentally by adapting the workflow proposed by Brown et al.^2^ During the initial process development, which predates Brown et al.,^2^ a solvent screen had been conducted by Takeda, and to avoid oiling out of the product, a methanol and water mixture was selected. The baseline process for the crystallization was to add seeds to a saturated solution with 10/1 (*v*/*v*) of methanol:water at55 °C followed by antisolvent (water) until reaching 10/6 (*v*/*v*) and finally cooling from 55 to 20 °C. The problem to be solved was that the isolated product was found to be heavily agglomerated, which increased the chance of impurity entrapment and could impact drug product manufacturability and performance.^3^

Thus, the purpose of the research described in this manuscript was to design an improved crystallization process to reduce the extent of agglomeration and obtain crystals with a regular shape, that is, an improved aspect ratio and lower degree of agglomeration, and a narrower size distribution.

A key theme of this paper is using PAT techniques to gain process understanding by interpreting the system in terms of nucleation, agglomeration, and growth behavior (see details in Section 3.1) throughout the crystallization process by tracking the solute concentration, solute mass, and supersaturation using UV/vis (the corresponding calibration model is discussed in Section S1). The premise is that product quality attributes are determined by the trajectory of the crystallization process within the phase diagram since the kinetics are directly related to the supersaturation profile;^2^ this paper provides a systematic exploration of the crystallization process, starting with making simple changes to the order of antisolvent addition/cooling stages and modifying the seed loading or seeding point to improve the product quality attributes. These effects alone are not sufficient to reduce agglomeration in the final product, and therefore more complex operations, such as wet-milling and feedback-enabled direct nucleation control, are applied to produce particle growth without further agglomeration.

There are different methods to control agglomeration, such as controlling the supersaturation within the metastable limit,^4^ adjusting crystallization trajectories crossing the liquid–liquid separation region by using a lower solute concentration,^5^ applying a bridging liquid to form spherical agglomeration to improve the size distribution and flowability,^6,7^ or changing the pH of the system.^8^ In addition, agglomeration and breakage are competing kinetic processes that occur simultaneously,^9^ so enhancing breakage is a potential way to reduce agglomeration and control the product size and shape.^10^ Ultrasonic devices are widely applied in lab-scale experiments to enhance the nucleation and reduce the agglomeration.^11,12^ Similarly, application of a high-shear rotor–stator wet mill during the crystallization process is an efficient method to reduce the crystal size,^13^ deagglomerate, and manipulate its shape, especially when needle-shaped crystals are formed.^14,15^ Crystal size reduction is caused by mechanical breakage,^13^ and needles are easily fractured to form short crystals and achieve a more uniform shape distribution.^16^ In this work, the effect of the supersaturation trajectories will be investigated. The methods of applying a bridging liquid and changing the pH are not suitable for this API product. Compared with high-shear stress mechanical devices,^17^ ultrasonic devices are restricted for scale-up.^18^ Therefore, wet milling was investigated as a means to achieve deagglomeration. There are two widely applied operation modes of a wet mill: (1) operating the wet mill at the end of the crystallization process,^19^ which can lead to production of fines that may adversely impact filtration, and (2) operating as part of the crystallization process, either post seeding,^20^ inserted at some point in the process,^21^ or at the end followed by a heat cycle.^22^

Undesired fine crystals generated by wet milling can be dissolved by subsequent application of temperature cycling, which can be controlled either via pre-defined (open-loop) temperature cycles^23^ or automatically determined via direct nucleation control (DNC) using closed-loop feedback control.^24^ DNC is a model-free feedback control method, which can automatically implement temperature cycles based on a desired FBRM target count.^25^ The effect of different temperature cycles will be discussed in the results, Sections 3.2 and 3.3.

## 2 Materials and Experimental Methods

### 2.1 Materials

The active pharmaceutical ingredient (API) applied in this study was compound X supplied by Takeda, USA (its molecular formula cannot be revealed for commercial reasons). The crystallization was intended to operate with an initial solvent volume ratio of methanol (purity of >99.5%, Fisher Chemical) to antisolvent water (deionized water) of 10/1 (*v*/*v*), and the final ratio was 10/6 (*v*/*v*). Further product information, such as solubility curves, were obtained from preliminary experiments. Background information supplied by Takeda, indicated that two polymorphs can be crystallized in the methanol–water system: form A (stable, rhombus morphology) and form L (metastable, needle morphology), which is known to transform to the more stable form A at room temperature. Form A API was supplied, as presented in Section S2, which shows agglomerated crystals with a mean size of *d*_4,3_ = 100 μm measured using a Malvern MasterSizer 2000.

**Figure 1:**
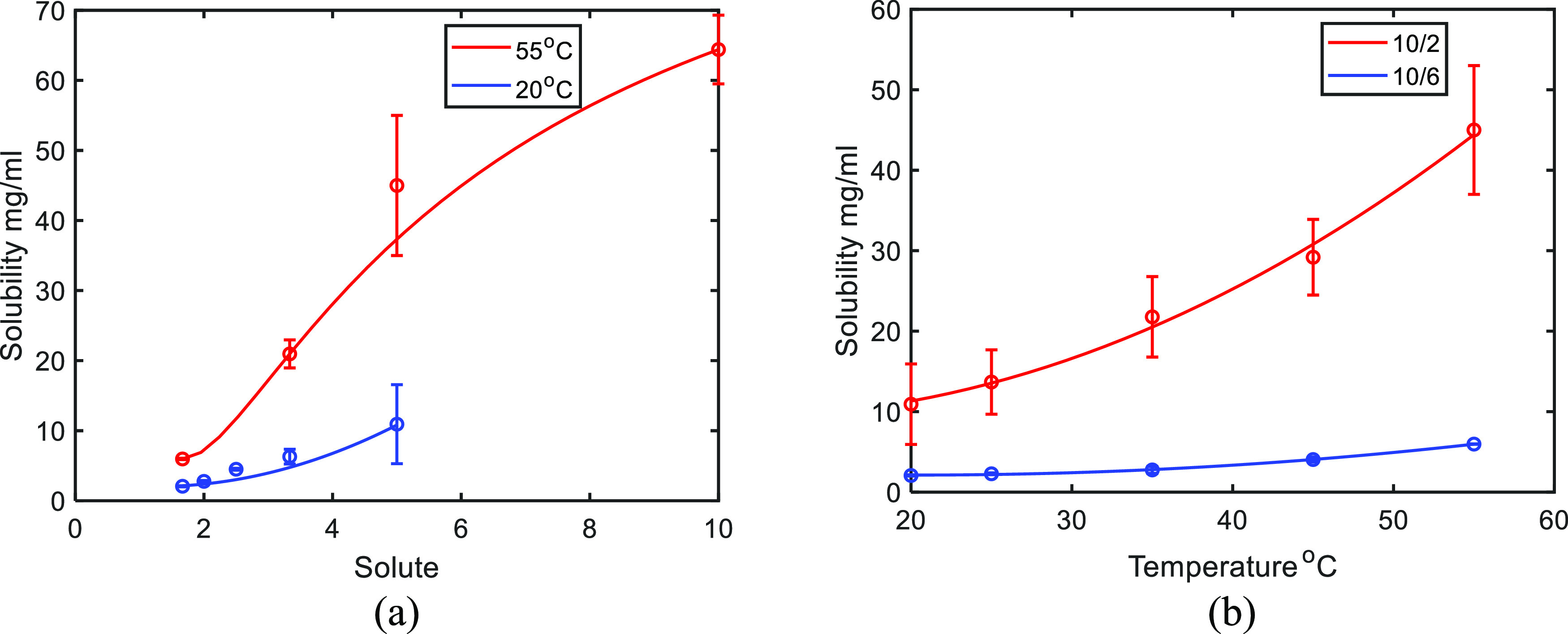
Solubility data for compound X: (a) as a function of the water (antisolvent) ratio of the total volume of the solution at two different temperatures and (b) as a function of temperature at two different solvent ratios (some error bars are too small to be visible).

A sequential antisolvent and cooling strategy was applied in this work to maximize recovery, so solubility experiments were separated into two parts: (1) the antisolvent addition process at constant temperature and (2) the cooling process with a constant solvent volume ratio. Solutions were prepared with an excess of solid compound X, held at prescribed values of the temperature and antisolvent ratio and allowed to equilibrate over 4 h. The solute concentration was obtained by gravimetric analysis of a filtered sample of the suspension, which was dried for 36 h in an oven at 40 °C (previously checked to be sufficient for complete drying; compound X does not form a hydrate or solvate). The corresponding solubility measurements are presented in Figure 1; each solubility measurement was repeated three times, and the corresponding error bars were calculated. The mass-based solubility results were converted to volume-based results by multiplying by the corresponding density. The details are presented in Section S3.

The solubility is more sensitive to changes in temperature at a higher ratio of methanol:water and has an order of magnitude-lower temperature gradient at a ratio of 10/6. This knowledge of the solubility data can be applied to determine the operating range of each crystallization step, as discussed in Section 2.3.

### 2.2 Experimental Setup

**Figure 2:**
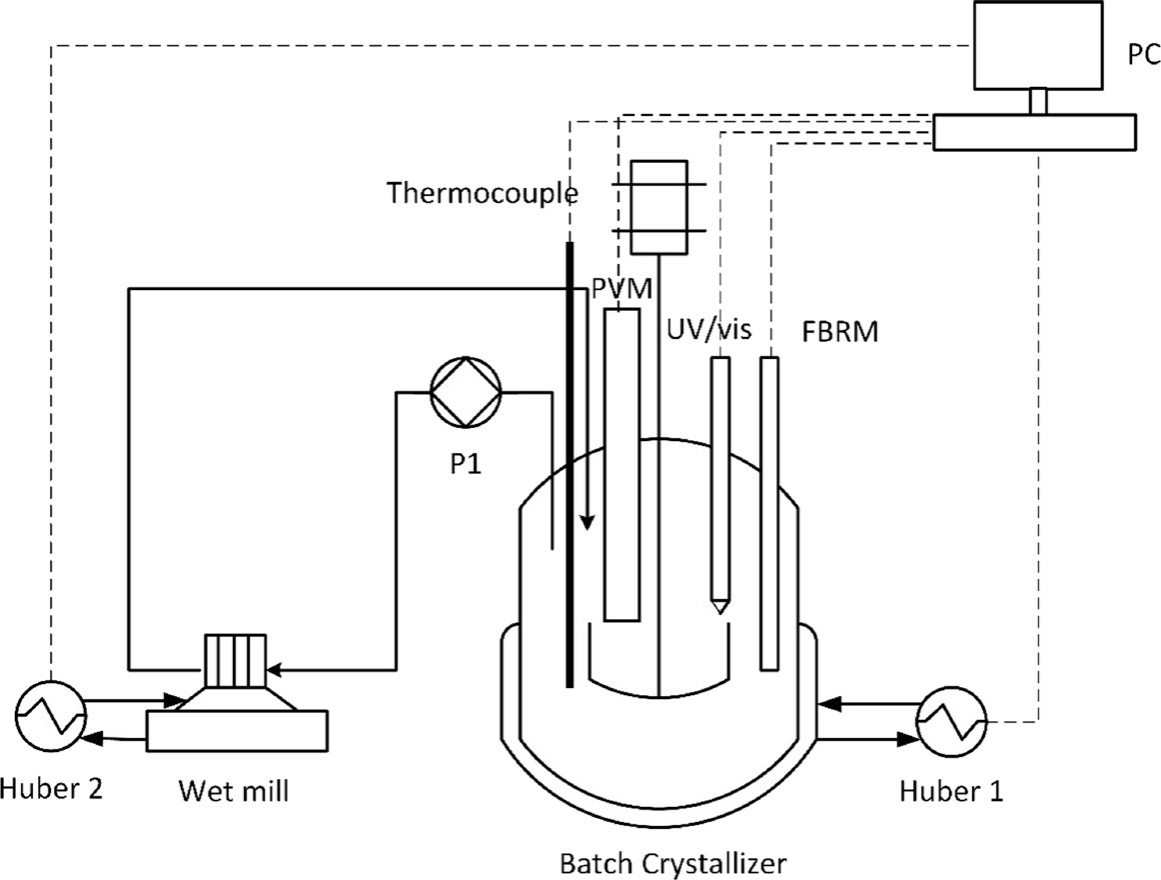
Schematic of the experimental setup.

The experimental rig was built using a batch crystallizer (500 mL) in combination with a wet mill setup in a recirculation loop, as presented in Figure 2.

The process temperature in the jacketed glass batch crystallizer was measured by a Pt100 thermocouple and controlled with a Huber recirculating heating/cooling bath (Ministat 125 with Pilot ONE, Huber). PAT tools were applied to monitor the process variables. A Lasentec D600L focused beam reflectance measurement (FBRM) probe (Mettler Toledo FBRM iC software, version 6.7.0) was used to measure the crystal chord length distribution (CLD) and number of crystals; the measurement time was set at 10 s. A V918 particle vision and measurement (PVM) probe (Mettler Toledo PVM image acquisition software, version 8.3), which is a high-resolution camera, was applied to record in situ crystal images. The solute concentration was monitored using the intensity of a specific peak in the spectra (see Section S1) measured by an ATR-UV/vis probe (MSC621 Carl Zeiss). The UV/vis measurement time was 5 s. All the process data were collected and controlled in a LabView-based software, Crystallization Monitoring and Control (CryMOCO).^26^ Slurry from the crystallizer was circulated by a peristaltic pump (P1) at 100 mL/min through an external loop containing the wet mill (IKA Magic Lab Module ULTRA-TURRAX). Dissipative heat is generated when the wet mill is operated, which can potentially dissolve the fine particles; the well mill is jacketed, and a second Huber was applied to maintain the temperature at the same value as in the crystallizer.

For offline measurements, laser diffraction (Malvern MasterSizer 2000 with Hydro 2000SM(A)) was used to measure the dried product crystal size distribution (water was applied as the dispersion solvent; the dispersion speed was 1500 rpm, and the obscuration range was 10–20%). Agglomeration was evaluated qualitatively using a microscope (GCT-20 Series Biological microscope, ASPEN). Finally, powder X-ray diffraction (PXRD) (Bruker D2 Phaser) was used to confirm the polymorphic form of the product.

In the experiments described in Section 3, it is challenging to interpret situations where size multiple-change mechanisms occur, such as growth, agglomeration, and breakage, which is why multiple sensors have been applied here. The depletion of the solute mass was monitored by UV/vis and is a direct indicator of crystal growth; agglomeration and deagglomeration can be qualitatively observed visually from PVM images and can be inferred from FBRM counts and chord length distributions; if there is limited secondary nucleation, then breakage can be inferred from the FBRM count increase and by the presence of fines in the PVM images.

### 2.3 Baseline Crystallization Experiments

**Figure 3:**
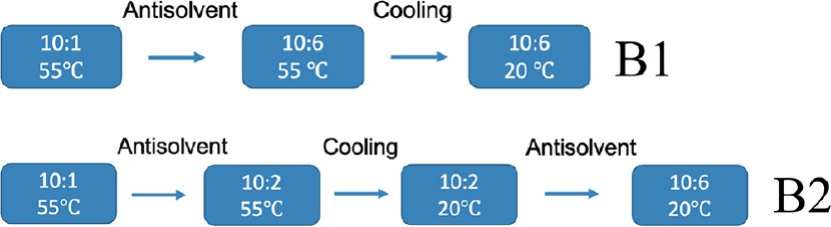
Two types of sequential antisolvent–cooling baseline methodologies B1 and B2.

Based on the solubility curves shown in Figure 1, there is a large decrease in solubility from the initial point where the solvent volumetric ratio is 10/1 at 55 °C to the end point where the solvent ratio is 10/6 at 20 °C so that a high yield can be achieved. To reduce the complexity of the operation, a sequential antisolvent–cooling operation was applied. Two baseline experiments with identical start and end conditions were investigated to study the effect of the operation order, as presented in Figure 3. For both processes, a saturated solution of the API in a 10/1 solvent at 55 °C was seeded and held for approximately 10 min before starting the first antisolvent addition. The order of the operations in B1 and B2 does not affect the overall yield of the process (assuming that the crystallization proceeds to equilibrium), but it does affect the kinetics and trajectory of the crystallization through the phase diagram.

In baseline sequence B1, the cooling process occurs at the lower methanol ratio, 10/6, whereas in baseline sequence B2, the cooling process takes place at a higher methanol ratio, 10/2. The solubility of the cooling stage in B1 is almost flat (Figure 1 shows a slope of 0.07 mg/(mL K) for the 10/6 solvent mixture in comparison to >0.8 mg/(mL K) at the higher methanol ratio). Thus, in B2, the supersaturation is mainly generated in the antisolvent addition stage. If wet milling and temperature cycling were implemented after the cooling stage of sequence B1, there will be limited capacity to dissolve the fine particles and grow larger crystals, which is shown in Section 3.2. Based on the trends of the solubility data shown in Figure 1, a temperature cycle after the cooling stage of B2 may have the potential to dissolve more fine particles and improve the product quality. The details of the corresponding temperature cycling DNC experiments are discussed in Section 3.3.

In this work, the final batch volume was set at 500 mL so that the initial volume of methanol was 312.5 mL and the volume of water was 31.25 mL. In experiments using sequences B1 and B2, the antisolvent addition and cooling rates were kept constant at 1 mL/min and either 0.2 or 0.5 ^°^C/min, respectively.

## 3 Results and Discussion

### 3.1 System Understanding

A set of baseline experiments (Table 1) was designed to understand the behavior of the system and especially to study crystal nucleation, growth, and agglomeration under different operating conditions.

**Table 1:** Process Conditions of Baseline Crystallization Experiments

exp	seeds (g)	type of baseline exp	seeding point	antisolvent and cooling rate
B1-1	6.87 (23.8%)	B1	*t* = 0 (10/1, 55 ^°^C)	antisolvent: 1 mL/min; cooling: 0.2 ^°^C/min for B2-1 and 0.5 ^°^C/min for other B2 experiments
B2-1	6.87 (23.8%)	B2		
B2-2	2.4 (9.8%)	B2		
B2-3	2.4 (9.8%)	B2	10/2, 55 ^°^C	

The % seed loading is defined as the mass of seeds divided by the total amount of API (seeds and in solution).

Experiments B1-1 and B2-1 were designed to compare the effect of the sequence of the antisolvent and cooling processes; the effect of seed loading can be compared using the results of B2-1 and B2-2. Experiment B2-3 was designed to investigate the effect of adding seeds later in the sequence, but before, primary nucleation would have occurred.

#### 3.1.1 Effect of Antisolvent and Cooling Order

**Figure 4:**
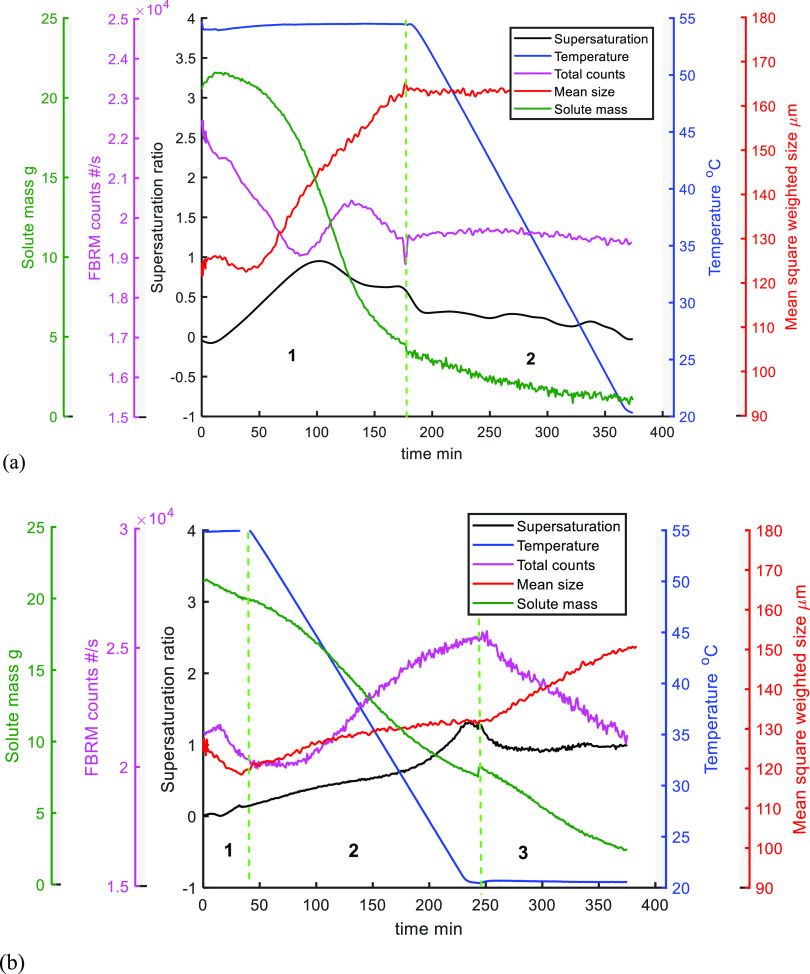
UV/vis measured solute concentration, calculated supersaturation, process temperature, FBRM counts, and mean square weighted size for (a) B1-1 (region 1 = antisolvent addition; region 2 = cooling) and (b) B2-1 (region 1 = 1st antisolvent addition; region 2 = cooling; and region 3 = 2nd antisolvent addition).

The process diagrams including FBRM counts, FBRM mean square weighted chord length (MSWCL), process temperature, solute mass, and UV/vis measured supersaturation are presented in Figure 4 for experiments B1-1 and B2-1, which investigated the order of the antisolvent addition and cooling stages. To interpret the effect of dilution and varying volumes due to the addition of water (antisolvent), the solute mass in solution is expressed as an absolute amount of compound X (gram). The supersaturation (*S*) is calculated from eq 1, and the data are presented in Figure 4.
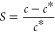
1where c* is the solubility and *c* is the solute concentration.

The calculation of *S* requires the solute concentration from UV/vis and the calibration model (Section S1) along with measurement of the process temperature and application of the curve-fitted solubility data. When the supersaturation is low, eq 1 shows that the calculation involves subtraction of two similar numbers, each with an associated error, and hence the estimated value of *S* is noisy (has a large relative error). For clarity of presentation, noise has been removed from the process variable records using a low-pass filter (Matlab function “low pass” with a sample time of 1 min and a normalized band-pass frequency of 10^–4^).

In both experiments, the seeds were added at the start and as expected, the solute mass decreased during the crystallization process, while the increase of the supersaturation ratio may be explained by the decrease of the solubility following antisolvent addition or cooling.

**Figure 5:**
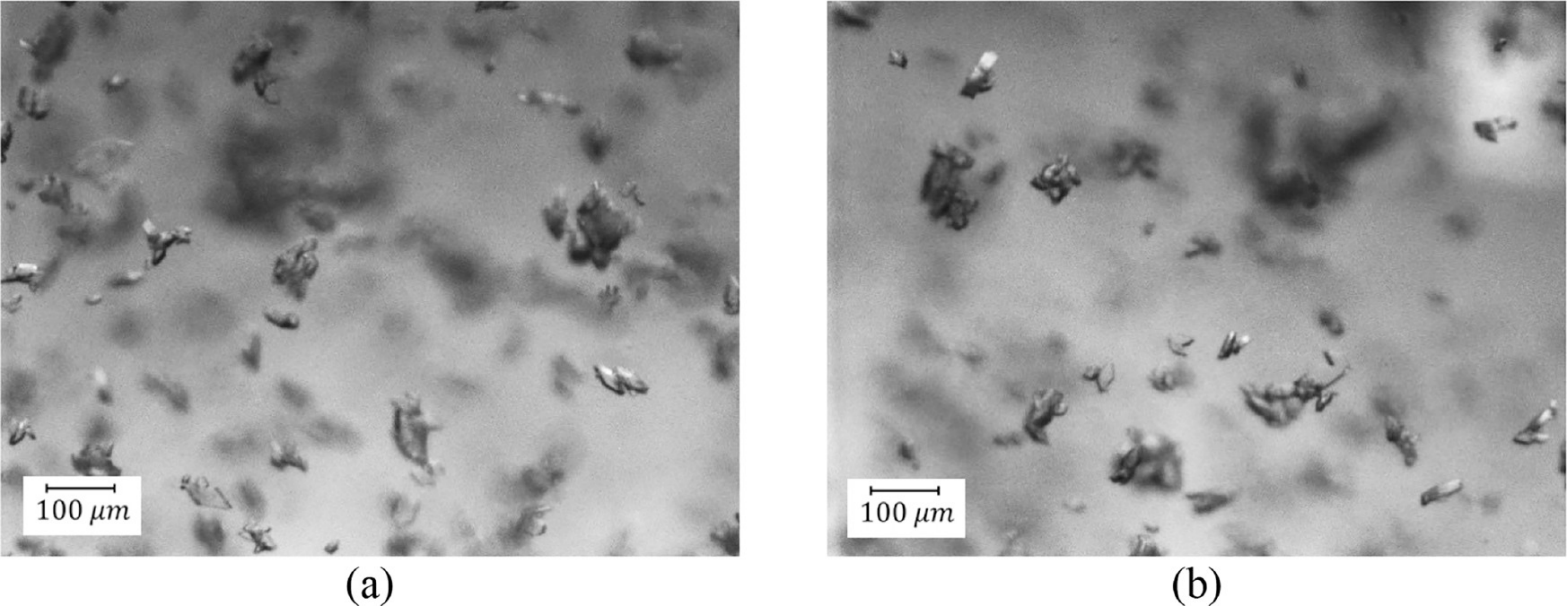
PVM pictures at the beginning of the process (after 2 min): (a) B1-1 and (b) B2-1 indicating agglomeration. Further PVM images for B1-1 and B2-1 are shown in Section S5.

For B1-1, the FBRM counts decreased slightly at the beginning of the first stage (0–40 min during the first antisolvent addition stage) due to agglomeration and dilution effects caused by the antisolvent addition. This agglomeration can be seen in the PVM recorded pictures, presented in Figure 5a and in Section S5. The supersaturation ratio at the beginning of region 1 in Figure 5a increased, and the solute mass in solution slightly decreased, indicating growth of the seeds, which is consistent with an increase of the FBRM mean size. The solute mass was very slowly consumed in region 2 (cooling process), and the FBRM mean size and counts remained almost constant. The increase of the supersaturation ratio in region 2 may be explained by reduction of the already very small values of the solubility during cooling (see Figure 2b). The absolute supersaturation was mainly generated in the antisolvent stage (region 1) so that the effect of the later cooling process is to produce only a small amount of crystal growth by the generation of limited supersaturation. This can also be seen by the consumption of the solute mass that decreased from 21 to 4 g during the antisolvent stage (region 1), compared to a change of only 3 g in the cooling stage (region 2). The system did not quite reach the equilibrium state because the holding time was not long enough at the lowest temperature (the supersaturation ratio was slightly greater than 0 (500 mL solution) at the end of the experiment).

For experiment B2-1, a similar trend was seen in the first region (1st antisolvent addition, Figure 4b) where there was a decrease of the FBRM mean size and counts, caused by dilution and agglomeration, as shown by the PVM images (Figure 5b). In region 2 (cooling process), growth and some secondary nucleation took place, so the mean size and FBRM counts increased. In region 3 (2nd antisolvent addition), the FBRM mean size increased more rapidly, and the solute mass fell, indicating growth. The decreasing of the counts was caused by a combination of dilution (due to the antisolvent addition) and possibly further agglomeration. The solute mass was consumed at approximately the same rate during the three stages of the process: 9 g of the solute mass was consumed during the first stage (1st antisolvent addition), 12 g during the second stage (cooling), and 7 g during the third (2nd antisolvent addition). These changes indicate the relative amounts of crystal growth and secondary nucleation that occurred at each of these stages.

**Figure 6:**
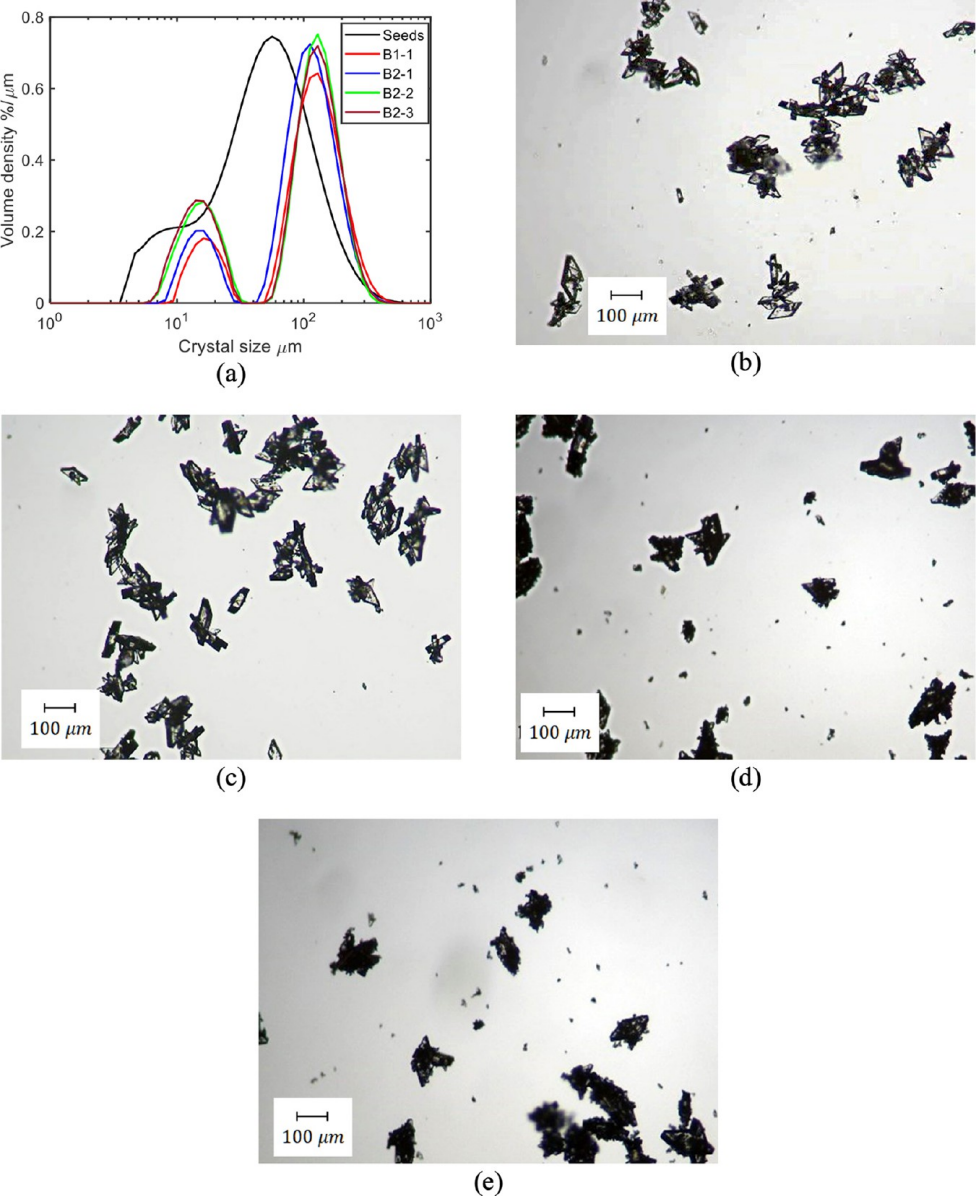
MasterSizer CSD measurements (a) and microscope pictures of the products from experiments of B1-1 (b), B2-1(c), B2-2 (d), and B2-3 (e).

Slightly smaller crystals were produced in the baseline experiment B2-1 due to the greater levels of secondary nucleation during the cooling stage. The final FBRM count of B2-1 is about 2.15 ×10^4^ /s, which is higher than in B1-1, 1.95 ×10^4^ /s. This is consistent with the offline MasterSizer measurements of the CSD and microscope images presented in Figure 6a–c.

The volume mean size (*d*_43_) for B1-1 was 144 μm, which is only slightly larger than the product size of 129 μm for B2-1. The volume mean size of the seeds was 100 μm.

Overall, the different order of antisolvent and cooling in B2 did not provide any substantial improvements (reduced agglomeration and more uniform CSD) in the product properties as similar mean size crystals were formed and the product was agglomerated in both cases.

#### 3.1.2 Effect of Seed Loading and Seeding Time

**Figure 7:**
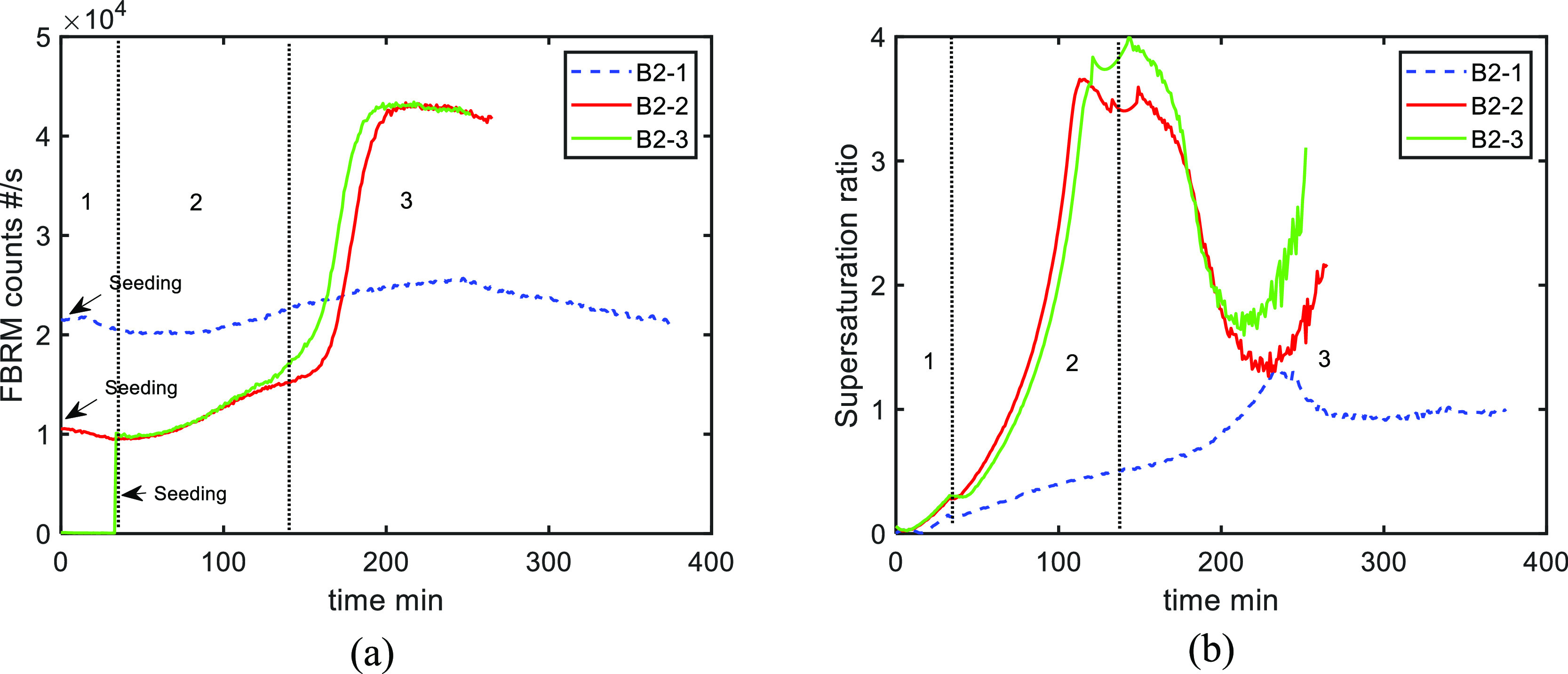
Comparison of experiments B2-1, B2-2, and B2-3: (a) FBRM counts and (b) supersaturation (the cooling rate in region 2 is smaller in B2-1, which is presented as dashed lines). The regions shown are only for experiments B2-2 and B2-3.

The next set of experiments aimed to study the effect of seed loading and seed time on the final crystal product qualities. The process diagram of B2-1 is shown in Figure 4b, and the results for B2-2 and B2-3 are shown in Figure 7. The impact of these process parameters on the supersaturation and FBRM counts is discussed next and compared to experiment B2-1. The variations of the other process variables for these experiments are presented in Section S4.

**Figure 8:**
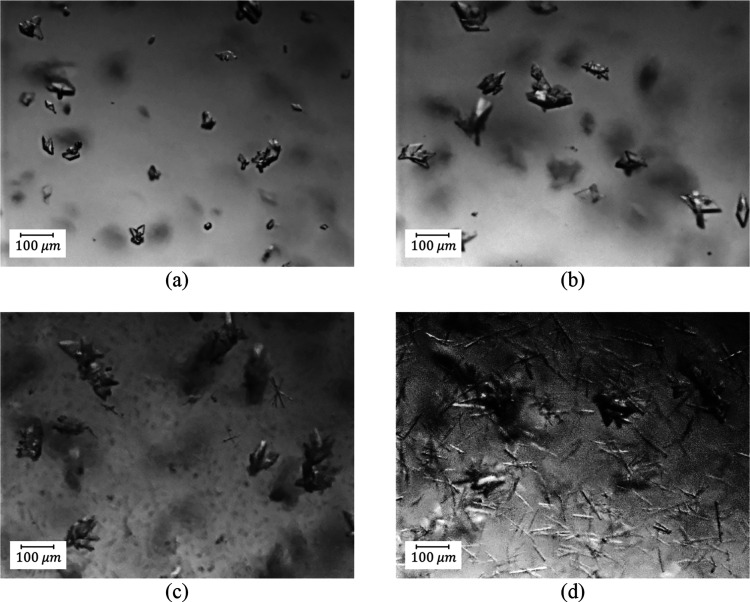
PVM pictures of B2-2: (a) seeds, (b) 2 min into the cooling stage (stage 2), (c) at the beginning of the second antisolvent addition (stage 3), showing the presence of needles (form L), (d) at the end of stage 2 (after 200 min), showing high levels of form L.

Experiment B2-2 was designed to investigate a lower seed loading (9.8%) compared with B2-1 (23.8%). The seeds were added at the beginning of the first stage of antisolvent addition, which was then followed by the cooling stage (sequence B2 in Figure 4). Figure 7 shows that both the supersaturation and FBRM counts increased continuously in regions 1 and 2 (1st antisolvent addition and cooling respectively). This indicates slow secondary nucleation and growth rates that do not fully consume the generated supersaturation by the end of the cooling process. In B2-2, 5 g of the solute mass was consumed by the end of stage 2 versus 21 g for B2-1, clearly demonstrating the impact of a lower seed loading. A rapid nucleation event leading to a spike in the FBRM counts can be seen at approximately 170 min (Figure 7b) during the second antisolvent addition (region 3). The corresponding PVM pictures show a noticeable change of crystal habit suggesting that polymorphic form L was nucleated (Figure 8). These needles appear to nucleate in the high-supersaturation region after the second solvent addition (region 3 on Figure 7b). The increase of the supersaturation at the end may be explained by the slow-growth behavior of both forms A and L.

**Figure 9:**
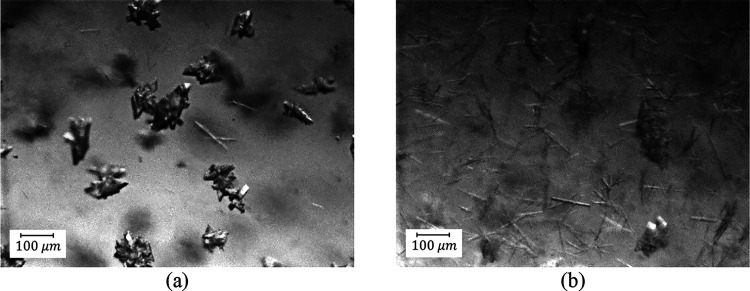
PVM pictures for experiment B2-3: (a) at the beginning of the second antisolvent addition stage where form L nucleated (after 130 min); (b) at the end of stage 2 (after 200 min), showing high levels of form L.

A similar effect was also observed in experiment B2-3 where seeds were added at the end of the first antisolvent stage (region 1 in Figure 7a) and held for 10 min before starting the cooling process (region 2). In the second antisolvent stage (region 3), the high supersaturation was consumed resulting in the nucleation and growth of form L crystals, as indicated by PVM pictures (see Figure 9). Again, there is a noticeable change of crystal habit from the rhombus morphology (form A) to needles (form L). These nucleation events during the second antisolvent addition produce a large number of small crystals, as shown by the FBRM data in Figure 7a.

**Figure 10:**
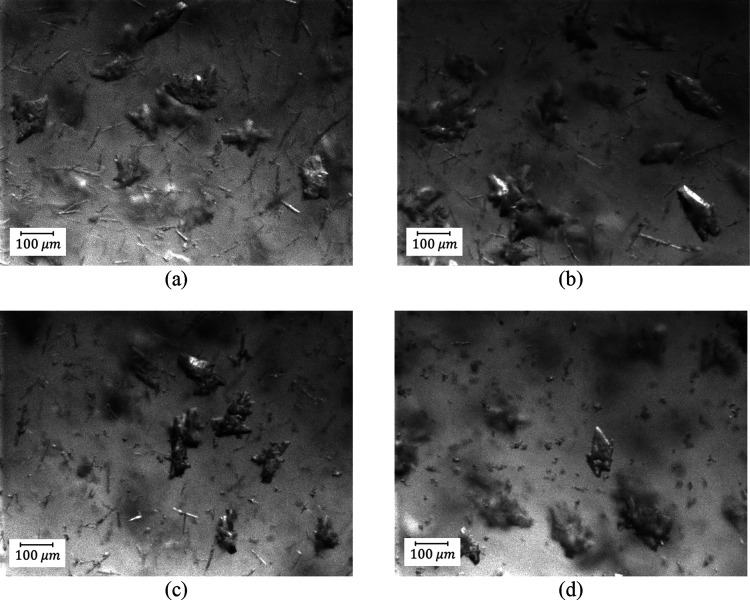
PVM pictures as a function of time for experiment B2-2 during prolonged aging at room temperature showing polymorph conversion to form A, which is illustrated by the disappearance of the needles: (a) 300, (b) 350, (c) 400, and (d) 450 min.

With a lower seed loading, mixtures of form A and form L were produced at the end of the crystallization process because of the higher levels of supersaturation generated during the process. However, form L is shown to undergo a polymorphic transformation to form A at room temperature within 3 h, as shown by the PVM images in Figure 10.

In Figure 6a, the CSD of the product crystals from B2-2 and B2-3 are almost overlapping, which means that the effect of delaying the seed addition point (at *S* = 0 in B2-2 and at *S* = 0.28 in B2-3) has a limited effect on the final product qualities. The dried products appeared to be agglomerated Form A crystals from both experiments and fine particles were generated by the transformation of form L shown in Figure 10a–d. Therefore, reducing the seed loading does not improve the crystal product quality.

#### 3.1.3 Conclusions from the Baseline Experiments

The baseline experiments were aimed to gain better understanding of the behavior of the system in terms of the supersaturation variations and to reduce the degree of agglomeration by changing the order of the antisolvent and cooling stages as well as investigating some key process parameters, namely, seed loading and seeding time. It was found that the stable form A is characterized by slow nucleation and growth kinetics, while the metastable form L can be easily nucleated at high-supersaturation conditions (supersaturation ratio of around 4). In order to control the form and minimize the probability of the metastable form nucleating, a high seed loading is recommended that will provide enough surface area to ensure growth-dominated desupersaturation and maintain an overall low supersaturation; 6.8 g of seed loading will be applied in subsequent experiments. The rates of consumption of the solute concentration and hence supersaturations were found to depend on the order of the cooling and antisolvent stages, which in turn affect the relative kinetic rates of nucleation versus growth versus agglomeration, for example, if anti-solvent addition precedes cooling, high levels of nucleation will be observed. Therefore, all subsequent experiments will adopt the B2 sequence of steps. Nevertheless, changes to the anti-solvent addition–cooling sequences and the seeding conditions did not provide the required improvements in product quality.

The next step was to investigate more complex operations such as application of wet milling and temperature cycling. The use of wet milling to induce primary nucleation was investigated, but this did not improve the required product quality (details are presented in Section S6). The next section describes the use of a wet mill as a breakage device to produce deagglomeration followed by temperature cycling, which was aimed at dissolving fine particles and producing growth of crystals without reagglomeration.

### 3.2 Pre-defined Temperature Cycle after Wet Milling

The application of wet milling in the crystallization process for compound X reduced the mean size of the crystals (through a process of deagglomeration) but generated a large number of fines. The details of the effects of geometry and rotating speed of the wet mill are presented in Section S7. To remove fines from the system, the temperature may be increased so that the solution becomes undersaturated, resulting in dissolution of the undesired fine crystals. If nucleation can be controlled, a subsequent cooling stage will allow the remaining crystals to grow and the mean size to increase. The details of these experiments are shown in Table 2.

**Table 2:** Experiments Using Temperature Cycles after Wet Milling^a^

		temperature cycling conditions				
exp	baseline methodology	*T*_min_ (^°^C)	*T*_max_ (^°^C)	heating rate (^°^C/min)	cooling rate (^°^C/min)	DNC set point (#/s)
TC1	B1	20	45	1	0.5	N/A
TC2	B2	20	50	1	0.2	N/A
DNC1	B2	20	50	1 (max)	0.3 (max)	37,000 ± 2000
DNC2	B2	20	50	1 (max)	0.3 (max)	35,000 ± 2000
DNC3	B2	20	50	1 (max)	0.3 (max)	30,000 ± 2000

a N/A = not applicable (open loop).

**Figure 11:**
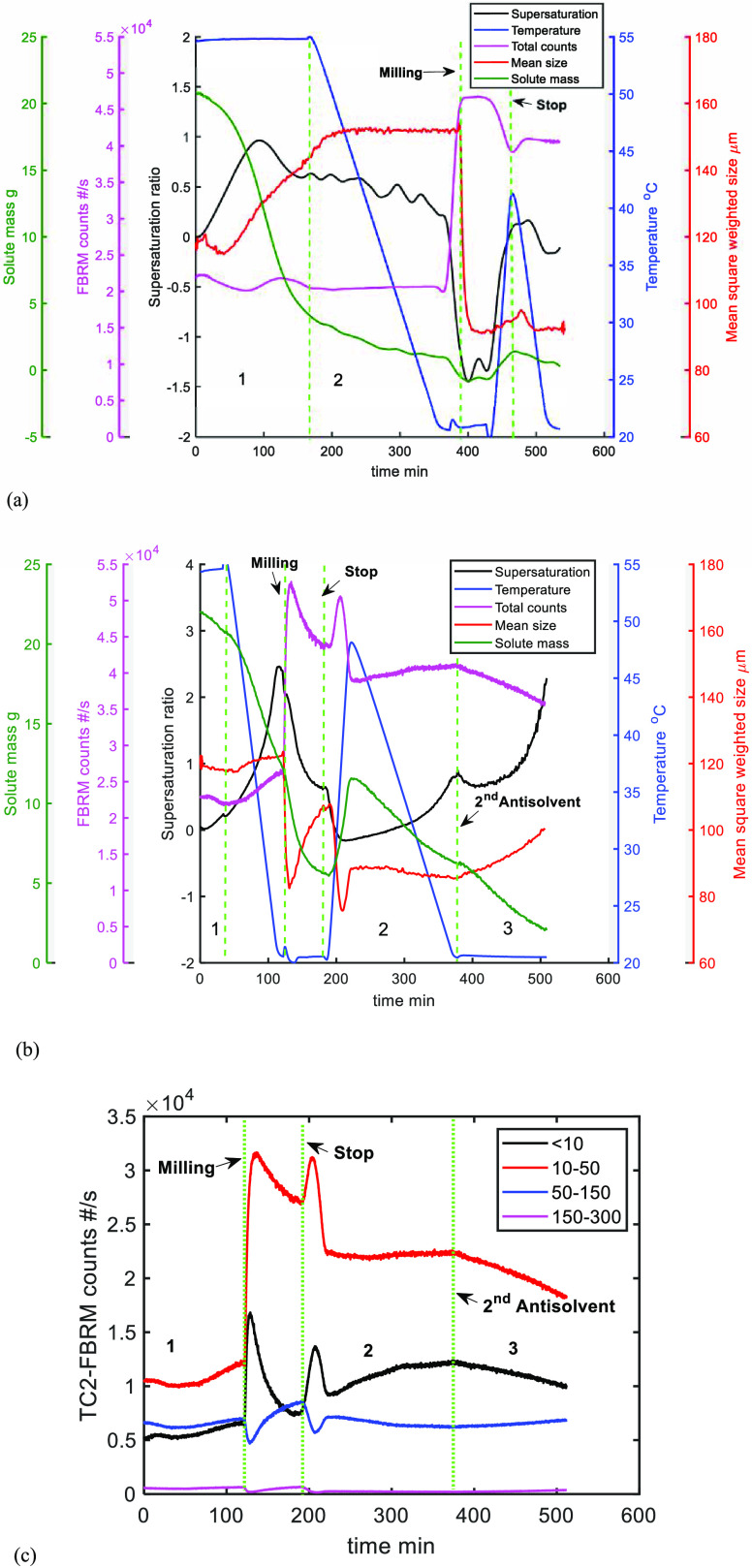
UV/vis measured solute concentration, calculated supersaturation ratio process temperature, FBRM counts, and mean square weighted size for (a) TC1 where the negative supersaturation during the wet milling period was caused by an error in the calibration models; (b) TC2 and (c) TC2 FBRM counts in chord length regions (μm).

The initial experiments used pre-determined (open loop) control of the heating and cooling steps using the temperature gradients and ranges specified in Table 2 (TC1 and TC2). The coarse mode rotor–stator with a rotational speed of 10,000 rpm was applied to reduce the crystal agglomeration, and the flow rate through the wet mill was set as 100 mL/min. The experiments for determining the operating parameters are presented in Section S7. The wet milling was operated after the cooling process, as shown in the process diagrams in Figure 11.

Milling was operated after the baseline sequence of B1-1 in TC1 (i.e., after anti-solvent addition), and as expected, the number of crystals immediately increased when the wet mill was started and leveled off reaching a steady state. The system was then heated from 20 to 45 ^°^C (jacket temperature) at a 1 ^°^C/min rate (the highest temperature of the process (41.5 ^°^C) was lower than 45 ^°^C due to heat loss). The number of crystals decreased during the heating period, and the mean chord length increased slightly, indicating that some dissolution had taken place. Some secondary nucleation occurred during the cooling stage of the temperature cycle because of the relative high supersaturation generated and poor growth kinetics of the system. However, the mean chord length did not change much, further suggesting limited growth. Furthermore, few crystals were dissolved due to the smaller solubility change over the range of the temperature cycle that effectively resulted in around 9% (2.5 g crystals were dissolved compared to ∼28 g of the total solute) of the material being redissolved. Overall, it may be concluded that the effect of one temperature cycle is limited after the initial cooling stage in the baseline experiment B1-1.

Experiment TC2 employed the conditions of baseline experiment B2-1, and the wet milling was operated for 50 min at the end of the cooling stage following the addition of the first portion of antisolvent to generate a 10/2 ratio of MeOH/H_2_O. A single open-loop temperature cycle was then implemented to dissolve the fine particles and redeposit the mass on the larger crystals. The corresponding process variables are presented in Figure 11b. Application of wet milling rapidly increased the FBRM counts through breakage, but then the fines were agglomerated (as found in experiments WT2 and WT3 and as presented in Section S7). The solute mass dropped because of the high surface area available for growth, and the mean chord length increased due to growth and agglomeration (at around 120 min). This is further evidenced in Figure 11c: the counts of the fine crystals (chord length ≤ 50 μm) initially increased because of the milling and then decreased due to agglomeration. As expected, the large chord lengths show the opposite trend. During the heating period (190 min), the fine crystals should dissolve and the total counts should decrease. Interestingly, the counts were observed to increase until ∼210 min, possibly as a result of the dissolution of the weak bridges connecting the fine and large crystals within the generated agglomerates that resulted in the release of the fine particles. Therefore, the count of fine particles increased initially and then decreased during heating (the number of large chord lengths (large crystals) show the opposite but self-consistent effects in Figure 11c). Slower secondary nucleation occurred during the cooling stage of the temperature cycle (220–370 min), so the FBRM counts slightly increased. The solute mass fell in this stage, indicating that crystal growth is occurring as well. At the end of the temperature cycle (∼390 min), the second antisolvent addition was made and the crystal mean chord length increased from approximately 80 to 105 μm, which is larger than the final mean chord length of TC1. Crystal growth in this final stage is evident since the solute mass continues to fall slowly, resulting in a build-up of supersaturation.

**Figure 12:**
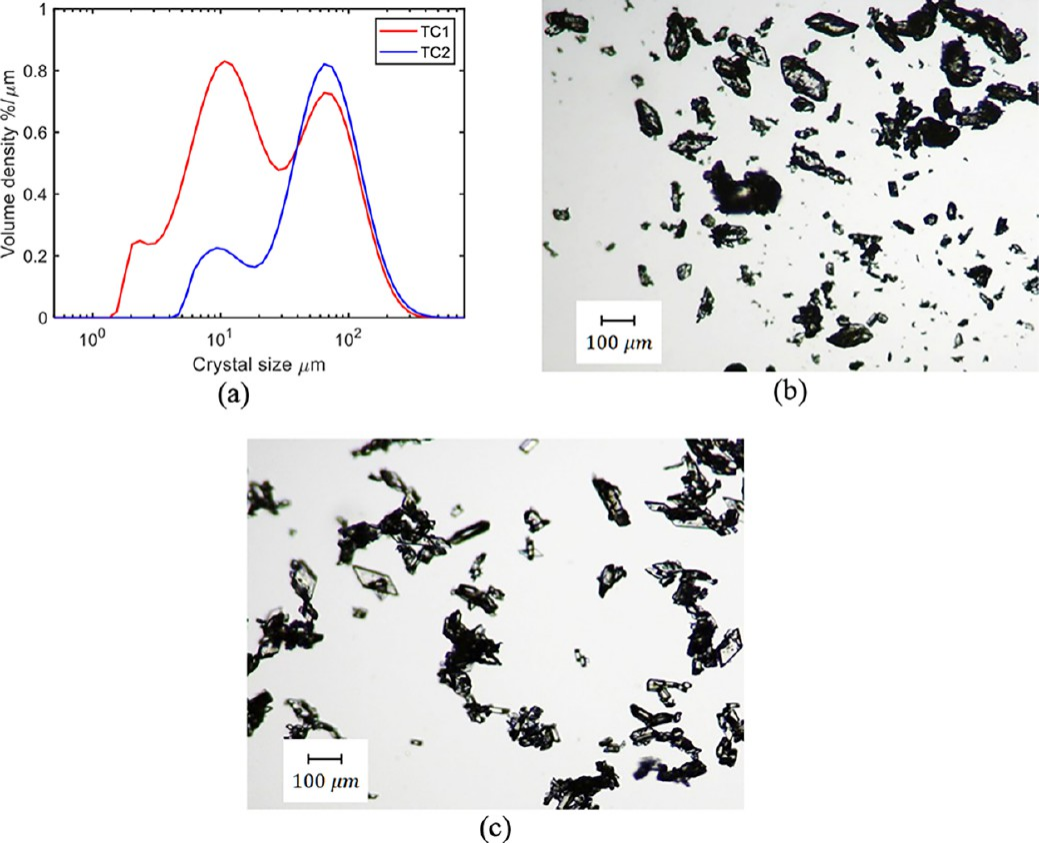
MasterSizer measurements (a) and product microscope images of TC1 (b) and TC2 (c).

Figure 12 shows the CSD measurements and microscopy images of the product; there are fewer fine particles in TC2 compared to TC1, indicating some improvement in product quality, but the product is still heavily agglomerated. Therefore, it is recommended to apply the temperature cycle to baseline experiment B2 to dissolve the fine particles generated by wet milling. Although the open-loop temperature profile can be pre-defined, it is not optimal in terms of the number of cycles, which depends on the maximum/minimum temperature or cooling rates being applied. DNC is an alternative method that uses feedback control from the FBRM as a sensor to define the number of temperature cycles based on the target crystal counts, and this is explored in Section 3.3.

Many previous studies have discussed the principle of temperature cycling to increase Ostwald ripening to remove fines and produce growth of larger crystals.^27^ It is well known that a single temperature cycle is not sufficient to manipulate the crystal size^28^ or crystal shape.^29^ The latter has also shown that the number of DNC cycles can be reduced by optimal design of the heat and cooling stages. Neugebauer et al.^30^ used 5–25 rapid temperature cycles (feasible in a small-scale tubular crystallizer) to demonstrate the feasibility of changing the crystal shape by altering the saturation trajectory alone. Similarly, Simone et al.^31^ showed that succinic acid crystals changed shape from plate- to diamond-like after multiple temperature cycling steps.

### 3.3 DNC Combined with Wet Milling

Direct nucleation control (DNC) is a model-free feedback control method used to reduce the width of a CSD, while increasing the mean crystal size.^25,27^ The method operates by applying heating and cooling cycles to control dissolution and nucleation rates to meet a set point, which is commonly a target number of FBRM counts.^27^ Boundaries are set on either side of the target. When the FBRM count exceeds the upper boundary, the maximum heating rate is applied; when the FBRM count is below the lower boundary, the maximum cooling rate is applied; and between the lower and upper boundaries, the temperature gradient for heating or cooling is set using proportional control.^25^

To apply the DNC, the first step is to determine the target counts and the boundaries. The counts of TC2 after the temperature cycle was about 4.1 ×10^4^/s, so the target count of the first trial was set at a lower value of 3.7 ×10^4^±2000/s (DNC1). The boundaries were set to approximately ±5% (2000/s). The target count was then reduced to 3.5 ×10^4^± 2000/s (DNC2) and 3.0 ×10^4^± 2000/s (DNC3) to improve product quality. The details of the experiments are summarized in Table 2.

**Figure 13:**
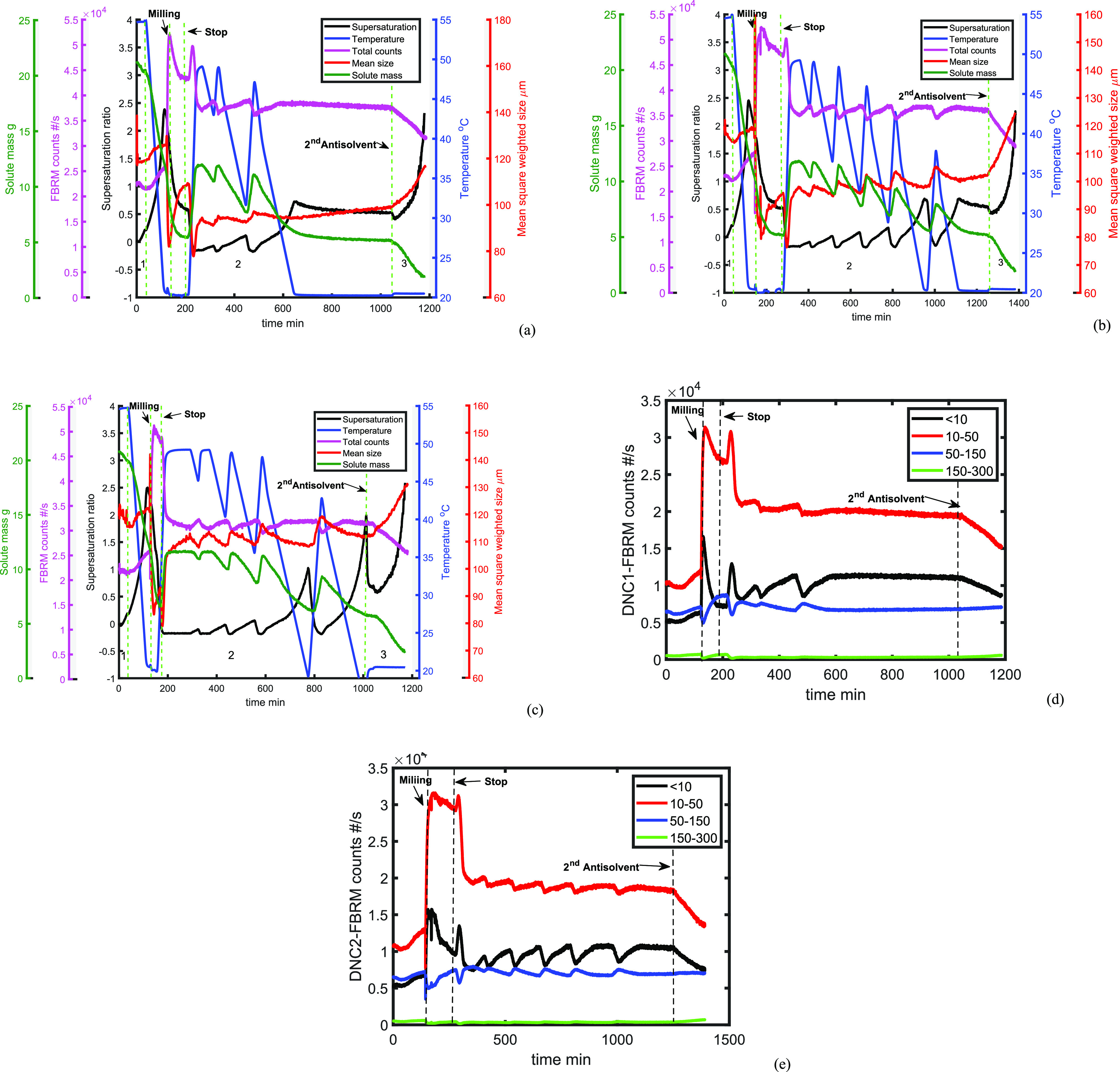
UV/vis measured solute concentration, calculated supersaturation, process temperature, FBRM counts, and mean square weighted size for (a) DNC, (b) DNC2, and (c) DNC3; (d) FBRM counts in different chord length ranges (μm) for DNC1 and (e) DNC2.

The corresponding process variables of each experiment are presented in Figure 13. The start of each experiment follows the operating sequence of TC2; wet milling is applied with the coarse mode geometry and 10,000 rpm for approximately 50 min after the end of the cooling stage at a solvent ratio of 10/2.

Generally, the variation of the counts in DNC1–3 follows a similar trend to that in TC2 during the milling stage and first heating cycle where agglomeration and deagglomeration took place (as described in Section 3.2). To demonstrate that agglomeration occurred in the milling stage, in DNC1, the system was held at 20 ^°^C for 20 min and the FBRM counts became stable, as shown in Figure 13a, at around 200 min. The supersaturation ratio was approximately constant during the same period. To reduce the agglomeration caused by the wet milling (see Figure 13d,e), a shorter milling time (25 min) was applied in DNC3 and the peak in the FBRM counts at the start of the first heating cycle (compare with DNC1 and DNC2, as presented in Figure 13d,e) disappeared, indicating that the release of fines from deagglomeration have been avoided.

The number of temperature cycles increased as the target counts decreased as it is commonly found; it takes longer to meet a more difficult target. Three cycles were applied in DNC1 and six cycles in DNC2 and DNC3 as determined by the feedback control loop. The heating time of the first heat period becomes longer to dissolve more particles with the decreased target counts, going from DNC1 to DNC2 and then DNC3. In some cases, the system is held at the highest temperature until reaching the target counts. To meet lower target counts and dissolve more particles, we would require the maximum temperature to be increased, but then this would take the system close to its saturation temperature for the starting solution (i.e., there would be a risk of complete dissolution).

**Figure 14:**
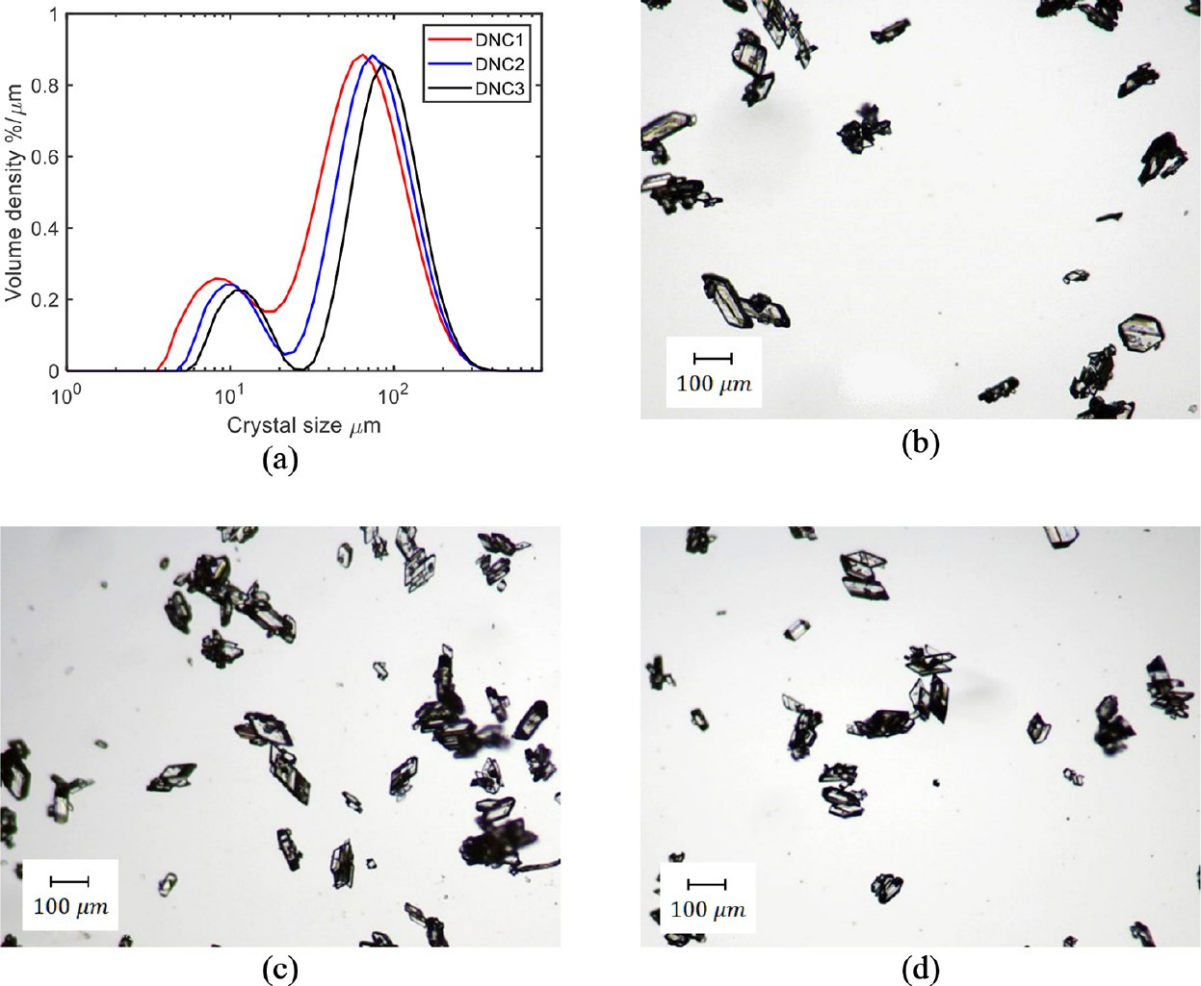
(a) MasterSizer measurements and product pictures for (b) DNC1, (c) DNC2, and (d) DNC3.

All DNC runs were able to hold the FBRM between the lower and upper bounds from cycle 1 onward, and there was growth of crystals and an increase in the mean size as indicated by the decrease of solute mass and increase of the MSWCL data. As the target counts were reduced, there was a modest increase in the MSWCL. As expected, the MSWCL was further increased after the DNC, through the addition of antisolvent (region 3). Reducing the target counts resulted in shifting the CSD to larger particle sizes, which is also shown in the offline measurements depicted in Figure 14.

**Figure 15:**
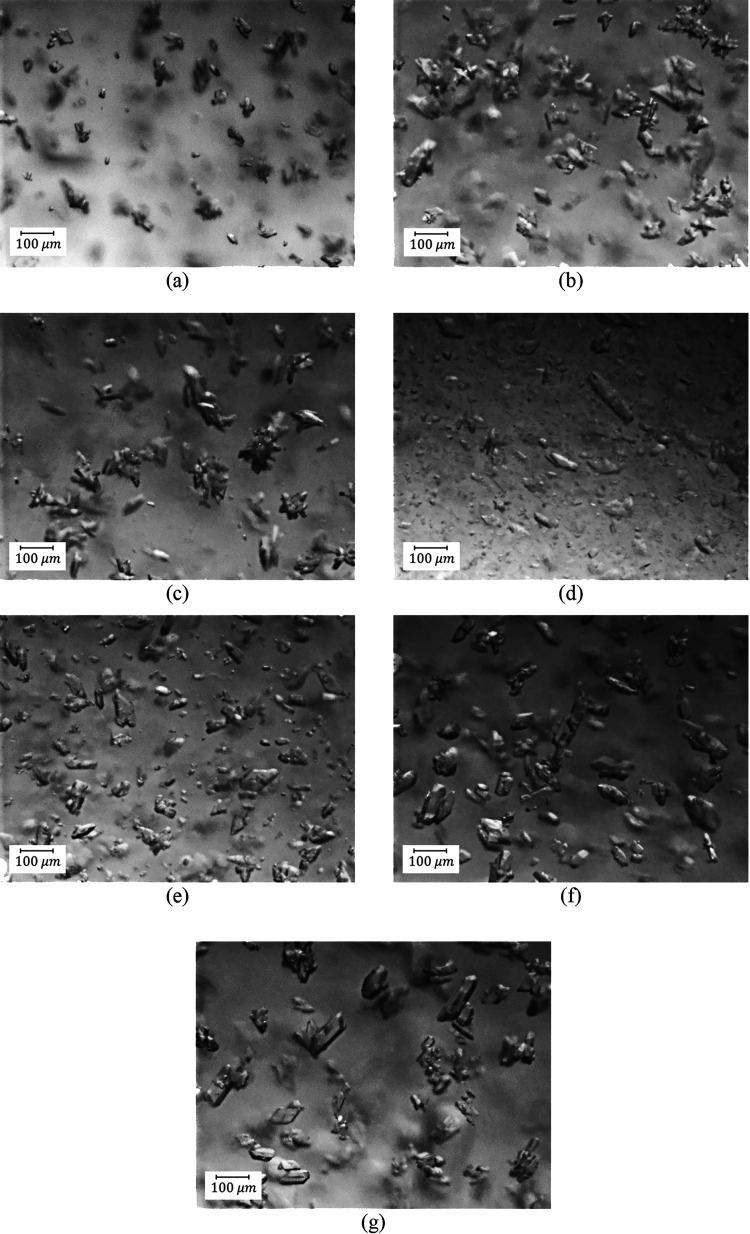
PVM pictures of DNC2: (a) seeds, (b) cooling stage (50 min), (c) start of milling (160 min), (d) during the milling (210 min), (e) DNC heating (300 min), (f) DNC cooling (600 min), and (g) final product (140 min).

The degree of agglomeration of product from these DNC runs is reduced compared to B2-1 based on the results shown in Figures 14 and 6b. Due to the properties of the system, some agglomeration can be observed during the second antisolvent step (region 3) where the FBRM counts decrease but the mean crystal size increases. Some agglomeration may be taking place during the filtration and drying processes, which precede optical microscopy. The benefits of the DNC approach can be clearly seen from PVM pictures shown in Figure 15 in the case of DNC2 as an example.

From the PVM images taken at different stages, the crystals were agglomerated after the first antisolvent stage and cooling stages (Figure 15b). When wet milling started, agglomerated crystals largely disappeared (Figure 15c,d) and lots of fine particles were generated. During the DNC stages, the fine crystals were gradually dissolved (Figure 15e), and individual crystals were observed after the last cooling cycle of the DNC (Figure 15f). Some agglomeration took place during the second antisolvent process (Figure 15g). However, overall, the agglomeration was reduced by using wet milling embedded within batch sequence B2 followed by feedback-controlled DNC.

In this work, DNC was only applied using heating and cooling to remove the fine crystals generated by wet milling, although it could also be applied in the antisolvent stage by switching the addition of the solvent/antisolvent when the solubility is insensitive to the temperature.^32^ With anti-solvent DNC, considerable dilution occurs, which can lead to low yields. In addition, supersaturation control (SSC) can also be applied in the antisolvent stage to maintain the low supersaturation for crystal growth.^26^ The optimized trajectories of antisolvent and temperature profiles can be simplified as pre-defined set points for guiding the scale-up process.^32^

Scale-up of the improved process is not considered here. It is known that (1) wet milling can be directly scaled up based on mechanistic models of particle breakage^33^ and (2) the time scale for heat transfer increases with the scale of operation; hence, DNC cycle times become significantly longer, so it becomes increasingly important to minimize the number of cycles required to achieve the required product quality.

## 4 Conclusions

The sequential antisolvent–cooling batch crystallization processes for compound X was studied using PAT tools to monitor the process variables and allow nucleation, growth, agglomeration, and breakage to be interpreted from the solute mass and supersaturation profiles. A UV/vis calibration model was constructed to estimate the solute concentration (see Section S1), which allowed supersaturation levels to be calculated throughout the batch process. FBRM was used to monitor the particle count per second and give an indication of particle size.

The methodology applied in this paper follows the general workflow for designing a crystallization process described by Brown et al.^2^ but extends it significantly to a more complex case involving the generation of supersaturation by a combination of antisolvent addition and cooling. Furthermore, the paper addresses the complexities of a real API system (Takeda compound X), which exhibits a strong tendency to agglomerate and forms an undesired polymorph at high supersaturation ratios; these effects can lead to poor quality of product crystals, e.g., needles and/or agglomerates with poor filtration, drying, and flow characteristics. The desired polymorphic form A was found to have a long induction time (particularly at low supersaturation) and then exhibit slow growth behavior, which indicated that too rapid changes in solubility conditions would lead to excessive supersaturation and undesired product characteristics. The proposed solution to manufacture improved quality of crystalline products was based on the fundamental understanding of the trajectory of the process through the phase (or solubility) diagrams to manipulate the supersaturation time history and control the relative rates of nucleation, growth, and agglomeration while avoiding the high supersaturations (by adding a higher seed loading), which lead to this undesired polymorphic transformation. It is shown that the solubility data are key to understanding this process and were used to propose better sequences of operations and improved seeding conditions, but this still was not sufficient to reduce agglomeration in the final product. The use of a wet mill gives an additional degree of freedom to break any agglomerates that have formed but has the disadvantage of producing a wide CSD with an excessive number of fine particles, which can reagglomerate and detract from the final product quality. Open-loop temperature cycling shows limited improvement in terms of removal of fines, but the process is not easily optimized and does not adapt to current conditions in the crystallizer. However, use of a closed feedback loop of DNC cycles based on a pre-determined FBRM target count may be more effective to remove fines by selective dissolution and grow larger particles without encountering further agglomeration. Thus, with redesign of the process, the product shows a much lower degree of agglomeration when manufactured using the improved sequence of antisolvent addition and cooling followed by a period of wet milling for deagglomeration and finally application of DNC to achieve a high yield of large single crystals of the desired polymorph.

## Supporting Information Available

UV/vis calibration results, image of the raw API material, solubility calculation, process diagrams for B2-2 and B2-3, PVM pictures of experiments B1-1 and B2-1, wet milling-induced primary nucleation, effects of rotor–stator geometry and rotational speed of wet milling, and dynamic vapor sorption of form A (PDF)

## Abbreviations

API: active pharmaceutical ingredient
CLD: chord length distribution
CSD: crystal size distribution
DNC: direct nucleation control
FBRM: focused beam reflectance measurement
MSWCL: mean square weighted chord length
PAT: process analytical technology
PVM: particle vision and measurement
PXRD: power X-ray diffraction
*a_i_*: fitted coefficients
*c*: solute concentration (mg/mL)
*c**: solubility (mg/mL)
*p*: selected UV/vis peak intensity
*S*: supersaturation ratio
*T*: process temperature (^°^C)
