# Supplementary material for: Use of Wet Milling Combined with Temperature Cycling to Minimize Crystal Agglomeration in a Sequential Antisolvent–Cooling Crystallization
Source: Cryst Growth Des. 2022 Jul 19;22(8):4730–44. doi: 10.1021/acs.cgd.1c01510 (PMC9354031; doi:10.1021/acs.cgd.1c01510)
Supplement: Supplementary file 1 — cg1c01510_si_001.pdf [file cg1c01510_si_001.pdf]

Supporting Information

Use of wet-milling combined with temperature cycling to minimize crystal agglomeration in a sequential antisolvent-cooling crystallization

Zhuang Sun[†], *Justin L. Quon*[‡], *Charles D. Papageorgiou*[‡], *Brahim Benyahia*[†], *Chris D. Rielly*[†*]

[†] Future Continuous Manufacturing and Advanced Crystallization (CMAC), Research Hub at the Department of Chemical Engineering, Loughborough University, Loughborough, Leicestershire, LE11 3TU, United Kingdom

[‡] Process Chemistry and Development, Takeda Pharmaceuticals International Company, 40 Landsdowne Street, Cambridge, Massachusetts 02139, United States

1. UV/vis calibration results

To track the variation of solute concentration, a UV/vis spectrometer was calibrated using known concentrations of active pharmaceutical ingredient (API), Compound X, over a range of temperatures and solvent ratios. The UV/vis absorption can be affected by the solvent ratio while the effect of temperature is limited (Billot et al., 2010). To capture solvent effects, two references were taken at solvent ratios of 10/1 and 10/6, respectively. The first calibration model (M1) is for an antisolvent process where the solvent ratio is changed to 10/1 to 10/6 at 55°C. The second calibration model (M2) is used to calculate the solute concentration during the cooling process from 55 to 20°C with a solvent ratio of 10/6. The last model (M3) is built for the same cooling process, with a solvent ratio is 10/2. The details of the experimental concentrations and operating conditions are listed in Table 1.

Table 1. UV/vis calibration experiments

Experiment	Solute concentration (mg/mL)	Temperature (℃)
M1-1	5.80-4.00	55.0
M1-2	8.40-5.90	55.0
M1-3	14.5-10.0	55.0
M1-4	34.9-24.0	55.0
M1-5	46.0-33.4	55.0
M1-6	48.0-33.7	55.0
M1-7	58.2-44.2	55.0
M2-1	4.00	54.7-20.5
M2-2	5.00	53.3-20.5
M2-3	6.00	54.4-20.4
M2-4	10.0	55.1-20.6
M2-5	12.1	55.1-35.4
M2-6	15.0	70.1-47.2
M2-7	19.0	54.9-48.3
M3-1	20.0	55.3-20.4
M3-2	30.0	55.6-20.3
M3-3	40.0	55.8-20.3

In each of the experiments above, a clear solution with known concentration was slowly cooled ($0.8℃/min$) or antisolvent was added ($1\ mL/min$) continuously until primary nucleation occurred and the corresponding collected spectra were applied to build the calibration model. For example, primary nucleation occurred at 48.3℃ in M2-7 so that spectra collected from 54.9 to 48.3℃ were used. The general form of the calibration model for constant temperature or constant solvent ratio is presented in eq. (1).

$$c = a_0 + a_1 x + a_2 p + a_3 xp \quad (1)$$

where c is the predicted solute concentration (mg/mL); a_i are the fitted coefficients; x is the absorbance of the selected peak and p is the process variable, which can be either solvent volume ratio for the antisolvent process (M1 series in Table 1) or the process temperature for the cooling stage (M2 and M3 series in Table 1).

The absorption peak is related to the absorbing wavelengths of the API molecule after removing the solvent reference (Rinnan et al., 2009). Therefore, the wavelength number of the selected peak is supposed to be the same for the different solvents. Here, first derivatives of the absorption coefficients from the spectra were used to remove the effect of the baseline shift during UV/vis measurements. A spectroscopic peak which is sensitive to the variation of process variables, solvent ratio and temperature, should be selected to build the model. Therefore, the selected wavelength number may be slightly different for different stages. In this work, the selected wavelength number of the derivative peak for M1 and M3 was 370 nm because the initial solvent ratio is close; for M2, wavelength number was 365nm, as presented in Figure 1.

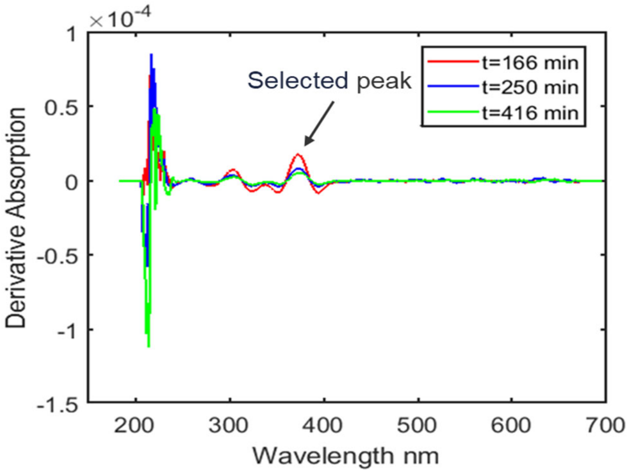

(a)

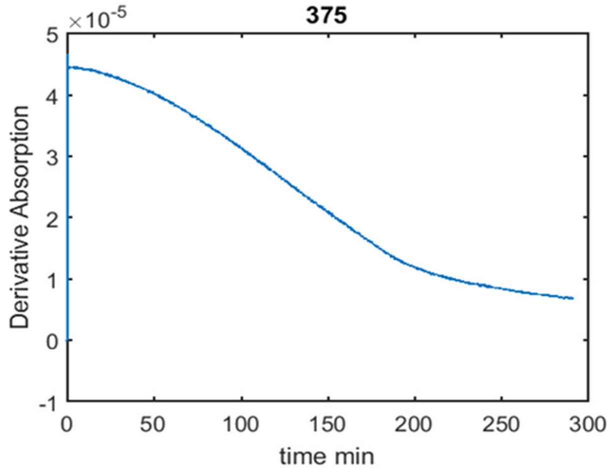

(b)

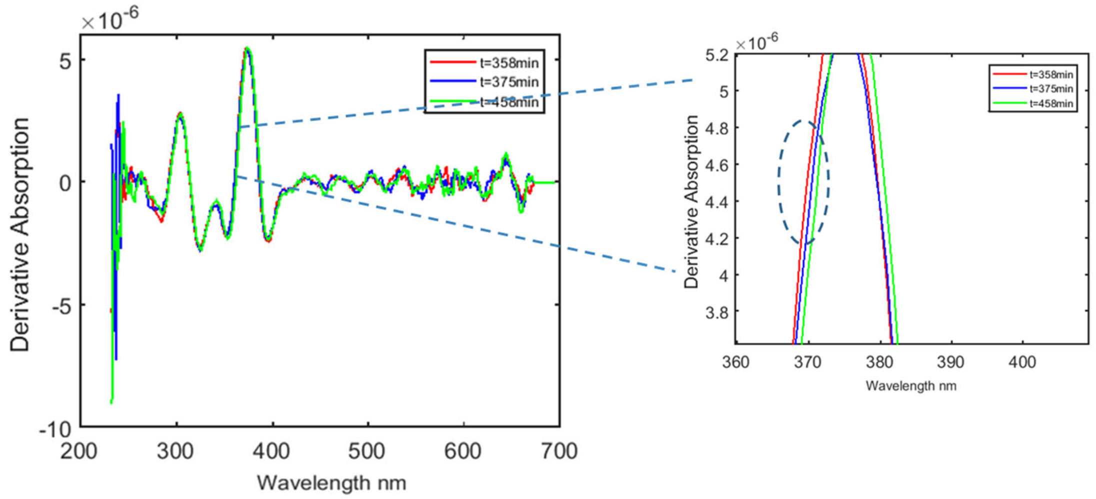

(c)

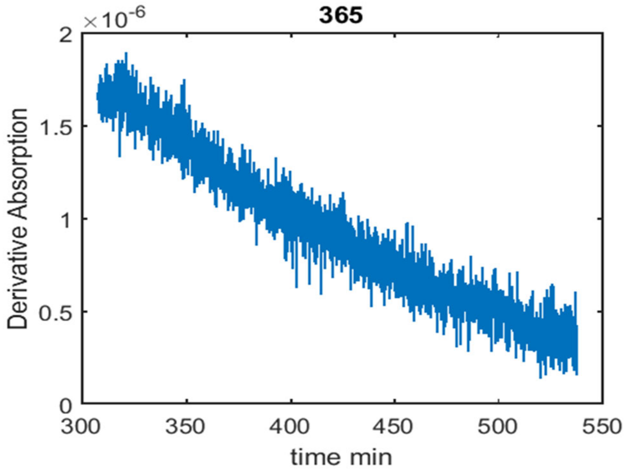

(d)

Figure 1. (a) UV/vis measurements at the different time for antisolvent process (M1 and M3); (b) selected UV/vis peak with time (the wavelength is 370 nm); (c) UV/vis measurement at different time for cooling process (M2); (d) UV/vis peak with time (the wavelength is 365nm).

Parts of the experiments summarized in Table 1 were used to identify the coefficients of the calibration model in eq. (1) and M1-2, M1-7, M2-3, M2-5 and M3-1 were applied for the validation for each model. Sampled data (1 minute) were applied and MATLAB solver 'fitnlm' was employed to fit the model.

The absolute relative error is applied to assess the validation performance, as shown in eq.(2).

$$error\ (\%) = \frac{|c_{cal} - c_{exp}|}{c_{exp}} \times 100\% \tag{2}$$

where c_{cal} is the calculated concentration by the calibration model and c_{exp} is the prepared known concentration for the calibration experiments.

The training fitting errors, and validation errors of calibration models are summarized in Table 2.

Table 2. Fitting and validation relative errors of the calibration models

Model	Fitting relative error, %	Validation error, %
M1	2.2	1.2 (M1-2), 3.2 (M1-7)
M2	4.2	5.4 (M2-3), 2.3 (M2-5)
M3	0.47	0.94 (M3-1)

The residuals of the validation results are presented in Figure 2. Generally, the absolute relative errors of the three models are all smaller than 6% which means the corresponding calibration models are reasonably accurate to calibration the process solute concentration. The residuals of M2 and M3 are almost evenly distributed between positive and negative values, while the

calibrated concentration seems higher than the actual data for M1, which is acceptable for the validation data.

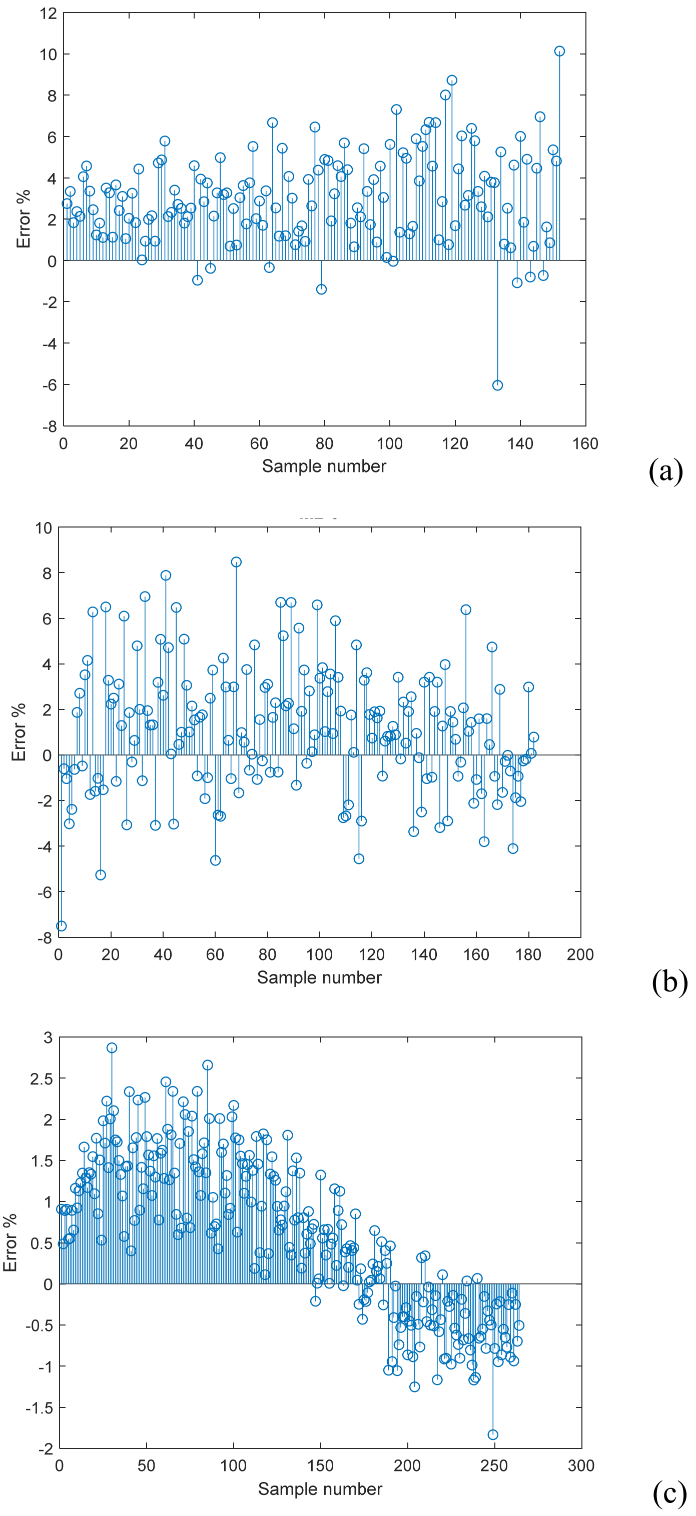

Figure 2. Residuals of the validation of each model: (a) M1; (b) M2 and (c) M3; the sample number is the spectra data collected during the cooling or antisolvent addition process.

2. Image of the raw API material

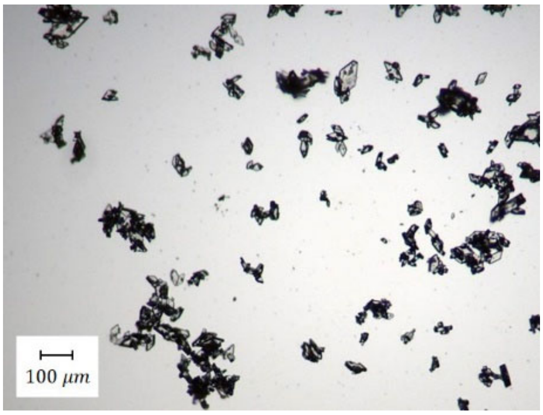

Figure 3. Microscope image of the raw API material.

3. Solubility calculation

The density of the mixed solvents at different temperature are presented in Table 3, which were applied to convert the mass solubility to volume solubility.

Table 3. Density of the mixed solvents at different temperature (mg/mL)

	55℃	45℃	35℃	25℃	20℃
Methanol	0.757	0.767	0.776	0.786	0.791
10/1(MeOH/Water)	0.779	0.788	0.796	0.805	0.810
10/2(MeOH/Water)	0.797	0.806	0.813	0.821	0.825
10/3(MeOH/Water)	0.813	0.821	0.827	0.835	0.839
10/4(MeOH/Water)	0.826	0.833	0.840	0.847	0.851
10/5(MeOH/Water)	0.838	0.845	0.851	0.857	0.861
10/6(MeOH/Water)	0.848	0.854	0.860	0.866	0.869

4. Process diagrams for B2-2 and B2-3

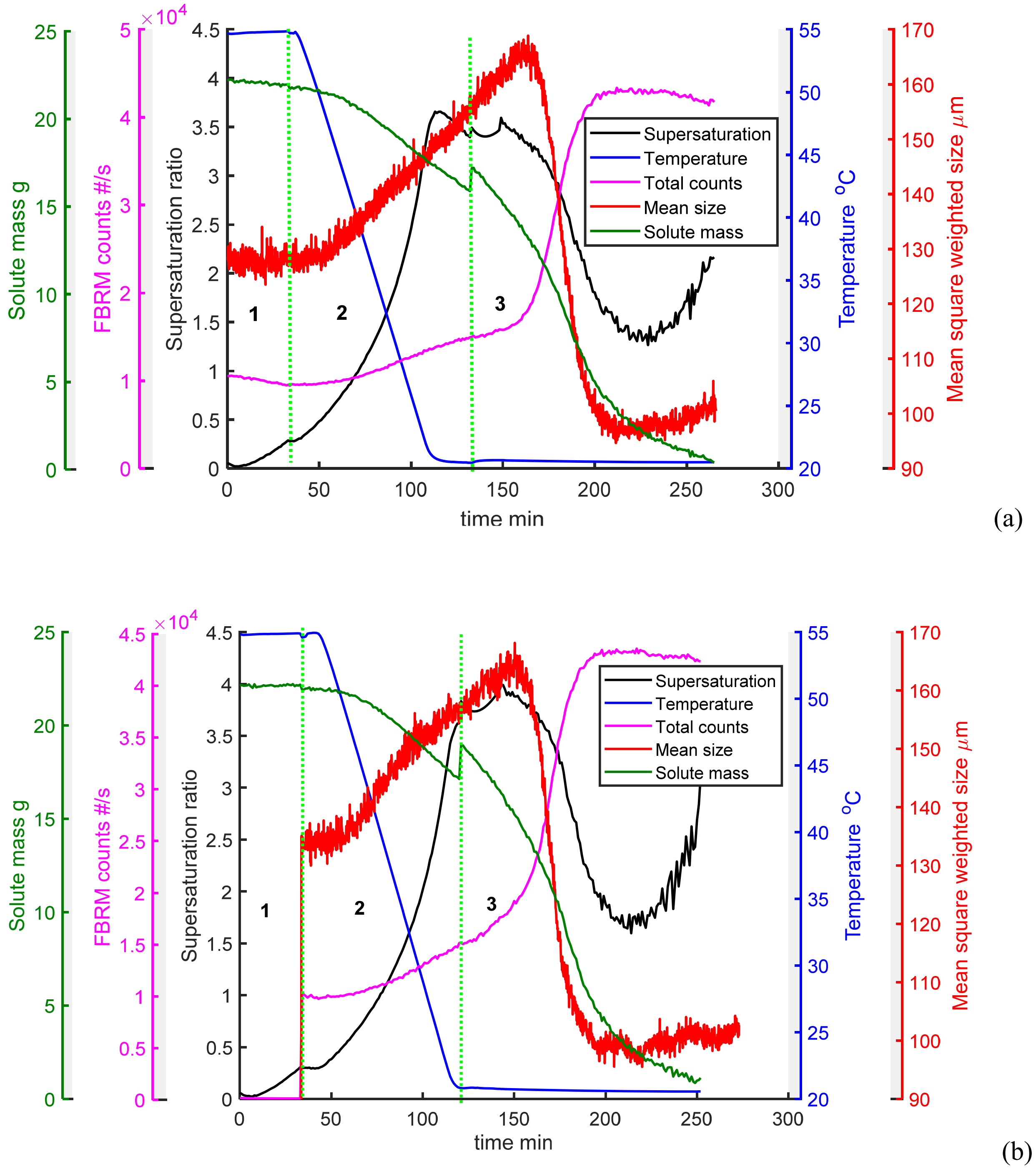

Figure 4. UV/vis measured solute concentration, calculated supersaturation, process temperature, FBRM counts and mean square weighted size for (a) B2-2 and (b) B2-3.

5. PVM pictures of experiment B1-1 and B2-1

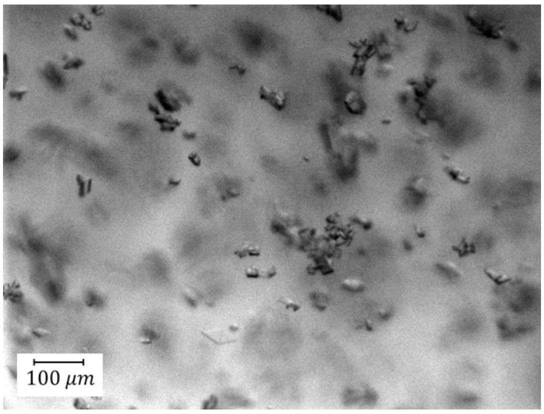

(a)

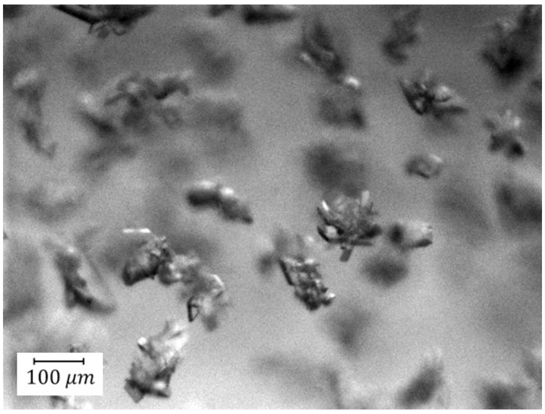

(b)

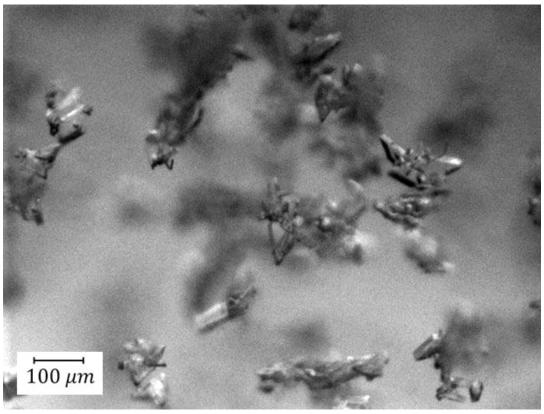

(c)

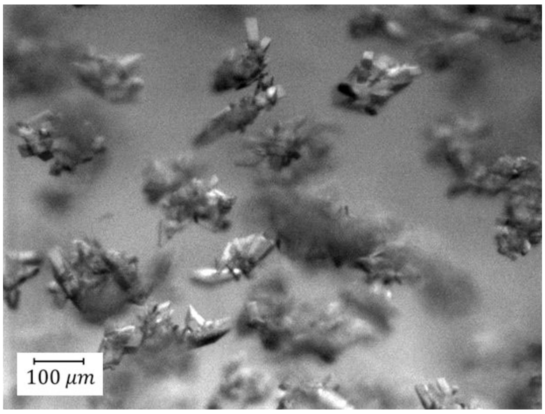

(d)

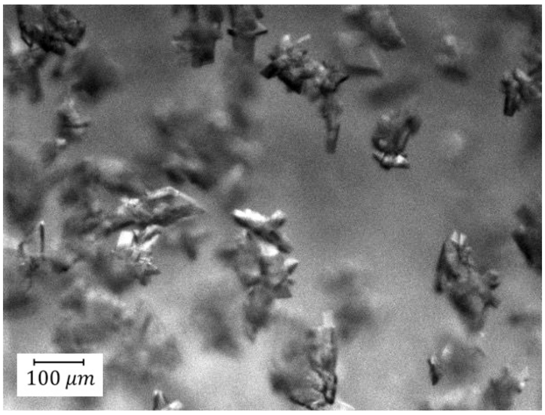

(e)

Figure 5. PVM pictures of B1-1: (a) seeds; (b) during the 1st region (antisolvent process); (c) at the end of the 1st region (antisolvent process); (d) during the 2nd region (cooling process); (e) at the end of the process.

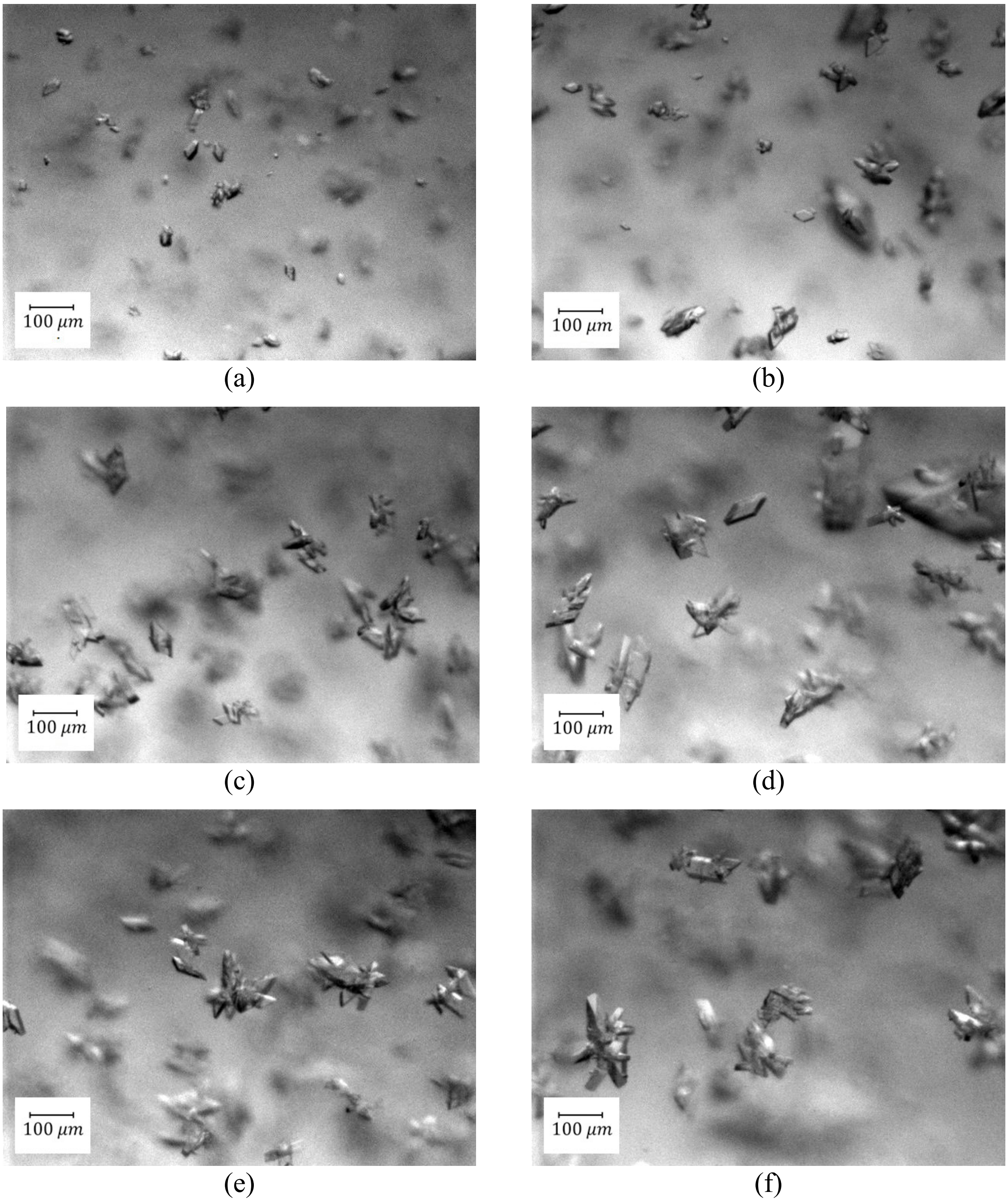

Figure 6. PVM pictures of B2-1: (a) seeds; (b) at the end of 1st region (1st antisolvent); (c) during the 2nd region (cooling); (d) at the end of 2nd region (cooling); (e) during the 3rd region (2nd antisolvent); (f) at the end of the process.

Seeds were agglomerated when the antisolvent process started in experiment B1-1 while the agglomeration was enhanced in 2nd antisolvent region in B2-1. Both of the experiments were ended with the heavily agglomerated crystals.

6. Wet milling induced primary nucleation

In the final baseline experiment B2-4, the batch crystallization was unseeded and primary nucleation was allowed to occur, making use of a shear induced mechanism (Yang et al., 2015) by circulating the crystallizer contents through the in-line wet mill. Figure 7 shows the results associated with this experiment. The objective was to determine whether primary nucleation could result in the generation of non-agglomerated material, that could then translate to an improved final morphology.

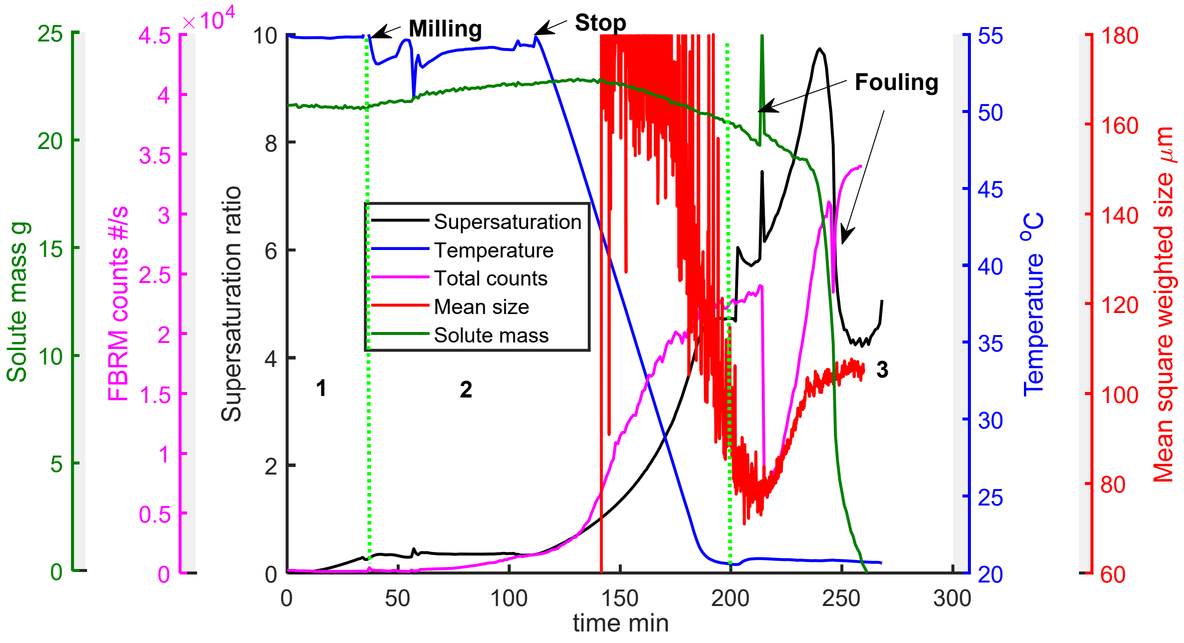

Figure 7. UV/vis measured solute concentration, calculated supersaturation, process temperature, FBRM counts and mean square weighted chord length (MSWCL) for experiment B2-4.

A small supersaturation ratio was generated during the first antisolvent addition (region 1), that did not lead to any nucleation. The wet mill was then started before the cooling stage (region 2) and held at 55°C for about 70 min. Even though the temperature of the wet mill was controlled by a second Huber whose set point was the same as the process temperature (55°C), the process temperature of the crystallizer was affected by the disturbances of the wet milling.

Primary nucleation occurred slowly after about 30 min of wet milling, and Form A was nucleated, as shown by the PVM (Figure 8a). As expected, the high shear rate generated by the wet mill enhanced nucleation. During the cooling stage (latter part of region 2), nucleation occurred leading to an increase in crystal counts, and supersaturation gradually built up resulting in fouling of the FBRM (necessitating periodic cleaning of the probe) and a high level of noise in the MSWCL trend. Growth rates were therefore too slow to consume the generated supersaturation, primary and secondary nucleation were prevalent and at the end of the cooling stage and the beginning of second antisolvent stage the product was heavily agglomerated (Figure 8b & c). In agreement with earlier observations for the low seed loading experiments (B2-2 and B2-3), the PVM images of Figure 8 (d) and (e) show that Form L nucleated during the second antisolvent stage, which started at a high supersaturation (at ~200 min in Figure *7*). This high supersaturation also led to fouling of the probes, which required periodic cleaning, as shown by the PVM image at the end of the experiment in Figure 8 (f), when the reactor contents were removed.

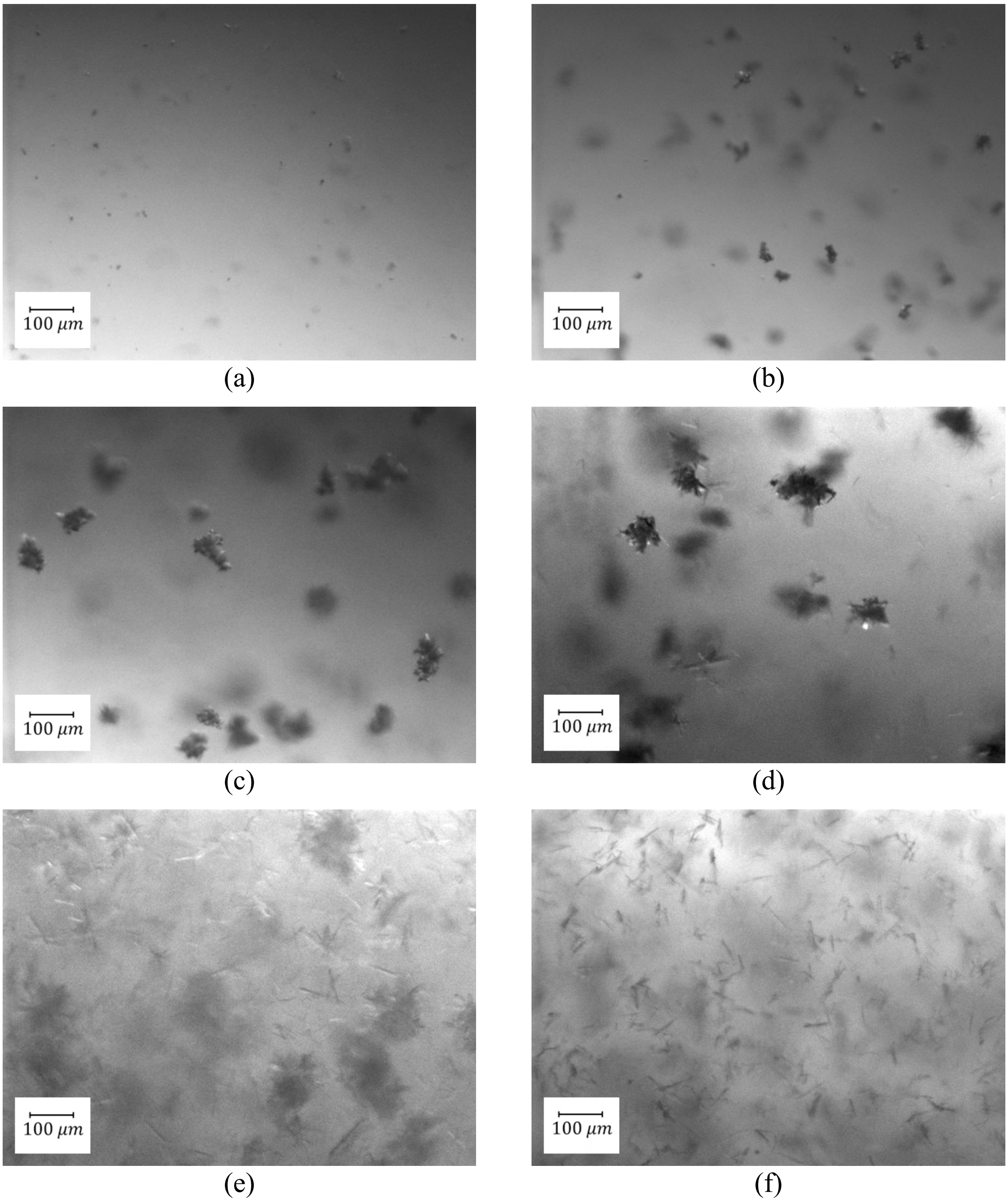

Figure 8. PVM pictures of experiment B2-4: (a) after wet milling–Form A (after 100 min); (b) at the cooling stage (Stage 2–after 160min); (c) at the beginning of the second antisolvent stage (Stage 3–after 200 min); (d) Form L nucleation in the second antisolvent stage (Stage 3–210 min); (e) growth of Form L in the second antisolvent stage (Stage 3–after 240 min); (f) PVM probe fouling when the crystallizer was empty at the end of the process–after 260 min.

The product crystals of B2-4 were dried before taking the PXRD measurements to assess their polymorphic form. However, as expected, the Form L crystals had transformed back to stable

Form A during drying and PXRD plots overlapped and showed no difference from the raw material, as shown in Figure 9 (a).

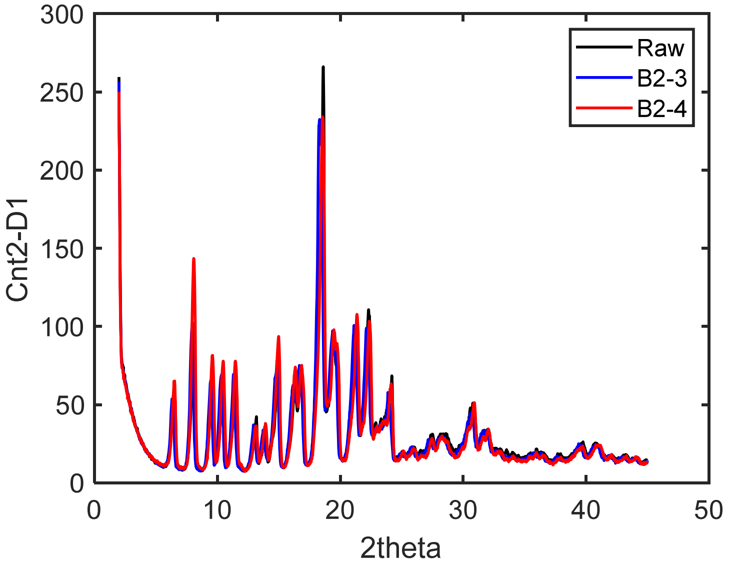

(a)

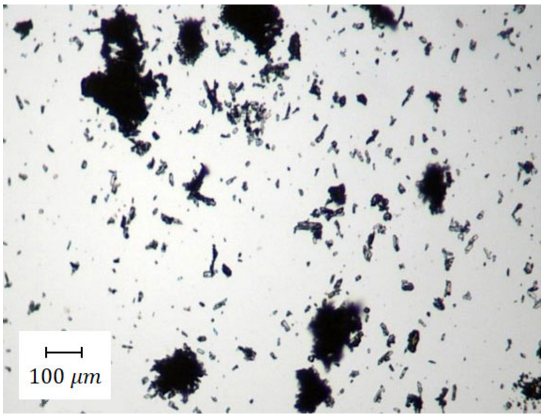

(b)

Figure 9. (a) PXRD results of seeds (raw material), B2-3 and B2-4;(b) microscopy picture of B2-4 product.

The offline microscope measurements (Figure 9b) presented similar trends, with more fine particles formed in B2-4 and with a high degree of agglomeration. Therefore, the use of wet-milling to produce shear-induced nucleation was not successful in reducing agglomeration and did not produce better quality final product. The high supersaturation generated during the second antisolvent stage, resulted in the nucleation of Form L as crystals which slowly converted to the desired Form A and agglomerated.

7. Effects of rotor-stator geometry and rotational speed of wet-mill

Two types of rotor-stator geometries were available, based on different number and size of teeth (coarse and fine, as shown in Figure 10).

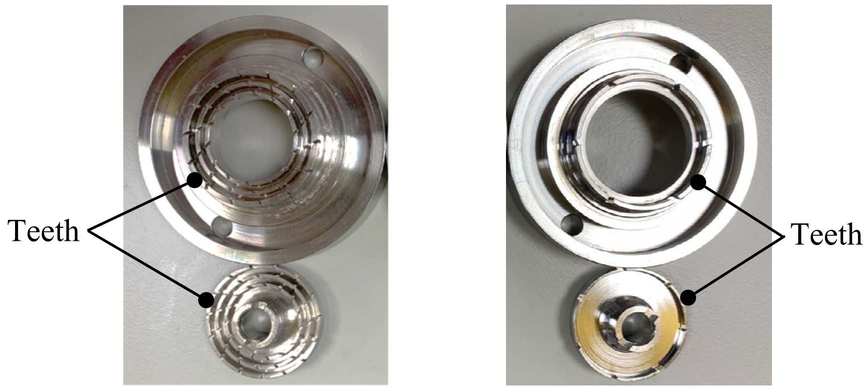

Fine mode *Coarse mode*

Figure 10. Fine (greater number of teeth) and coarse (fewer number of teeth) rotor-stator geometries

The performance of the wet mill as a breakage device depends on its design properties and operation mode (Özcan-Taşkin et al., 2011). The impact of different rotational speeds and rotor-stator geometries on particle morphology was investigated at the end of baseline experiment B1 (solvent ratio is 10/6 at 55°C) because of the high degree of agglomeration in the product material under these conditions. The FBRM counts measurements were used to check the performance of wet milling as a function of the different parameters with an increase in the rate of counts and decrease of the mean CLD being indicators of effective de-agglomeration. The corresponding experiments are summarized in Table 4.

Table 4. Effects of geometry and rotational speed of wet mill

Exp.	Baseline Methodology	Wet milling point	Geometry	Rotational speed (rpm)
WT1	B1	End of cooling	coarse	6000
WT2	B1	End of cooling	coarse	10000
WT3	B1	End of cooling	fine	6000

The performances of the coarse and fine geometries were compared to choose the most suitable configuration for the later deagglomeration studies. Different rotational speeds were also investigated, as indicated in Table 4. A 23.8% seed loading (6.87 g) was added at the start of all experiments, as discussed in the section 3.1 of the manuscript. The flow rate passing through the wet mill was set at 100 mL/min, which corresponds to near 10 crystallizer turnovers during the course of the experiment.

Comparisons of the corresponding process variables during the milling stage are presented in Figure 11. In each case, the process behavior should be the same up until the start of the wet-milling operation (~380 min following operating sequence B1) and any differences simply reflect small levels of variability in the system.

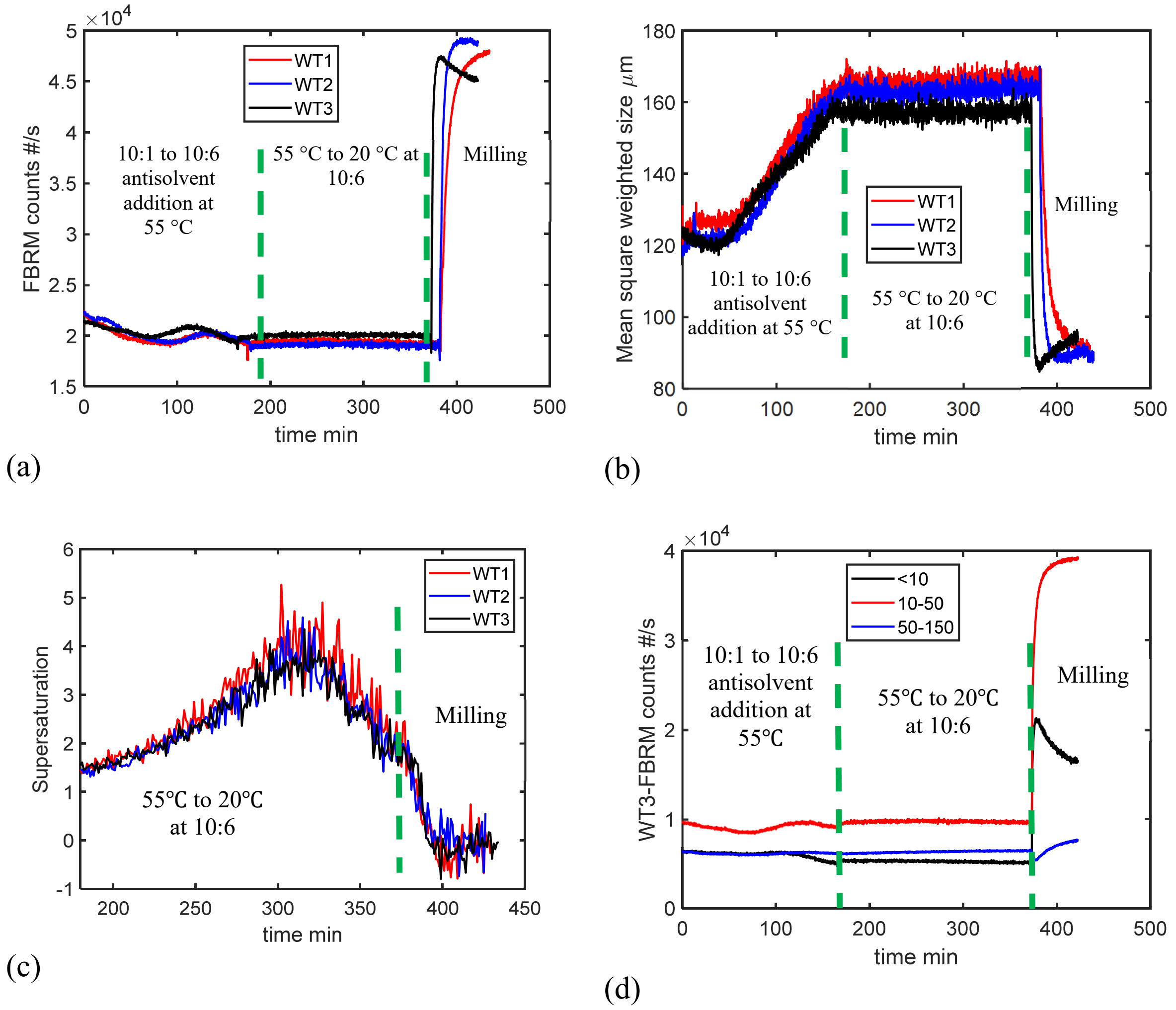

Figure 11. Process variables of experiments WT1, WT2 and WT3: (a) FBRM counts; (b) FBRM WMSCL; (c) UV/vis measured absolute supersaturation (started at cooling stage followed by wet milling stage); (d) FBRM counts for the different chord length ranges (μm) for WT3.

As expected, the higher rotational speed (WT2) increases the breakage rates and hence the FBRM crystal counts increased at a faster rate within the same wet milling time, as presented in Figure 11 (a). At the end of milling, the FBRM counts of WT1 were still increasing due to the limited milling time, while the counts of WT2 started decreasing, possibly caused by re-agglomeration. Breakage and agglomeration processes occur simultaneously inside the wet mill and within the volume of the crystallizer vessel. Higher rotational speeds increase the collision rate and possibly the surface energy of the particles which increases the agglomeration rate. The mean chord length data in Figure 11 (b) indicate that the crystal mean size in WT2

was slightly higher at the end of milling with almost zero supersaturation remaining (as shown in Figure 11 (c), the solubility of Compound X is constant during the milling period as the temperature and solvent composition was not changed), which would also indicate some agglomeration.

Form A crystals were produced and as noted in section 3.1 of the manuscript, the growth rate is slow and hence the solute consumption rate is low so that the supersaturation increased at the beginning of the cooling stage. More crystal surfaces were formed during the milling stage which accelerates the consumption rate through growth, so that the supersaturation decreased until reaching the equilibrium state (supersaturation became zero at the end of the wet milling operation).

Normally, breakage rates can be enhanced by selection of the fine rotor stator, when the same rotational speed and milling time are applied as a result of the smaller gap sizes of these rotor-stator geometries. However, in a system that is prone to agglomeration, these effects may be difficult to detect from CLD measurements. For WT3, which employed the fine rotor stator, the final decrease of total counts as presented in Figure 11 (a), is caused by agglomeration as shown in Figure 11 (d). After the initial breakage event, the counts of fine particles (smaller than 10 μm) were decreased, while the counts in the region of 50-150 μm were increased, which means the fine particles were agglomerating. Therefore, the mean chord length was increased towards the end of the milling stage, as shown in Figure 11 (b). The consumption of the supersaturation presents a similar trend to other wet milling experiments. Greater surface areas generated by wet milling can accelerate the solute consumption rate in a slow-growth rate system.

The MasterSizer measurements and dried product microscope images from the above experiments are shown in Figure 12.

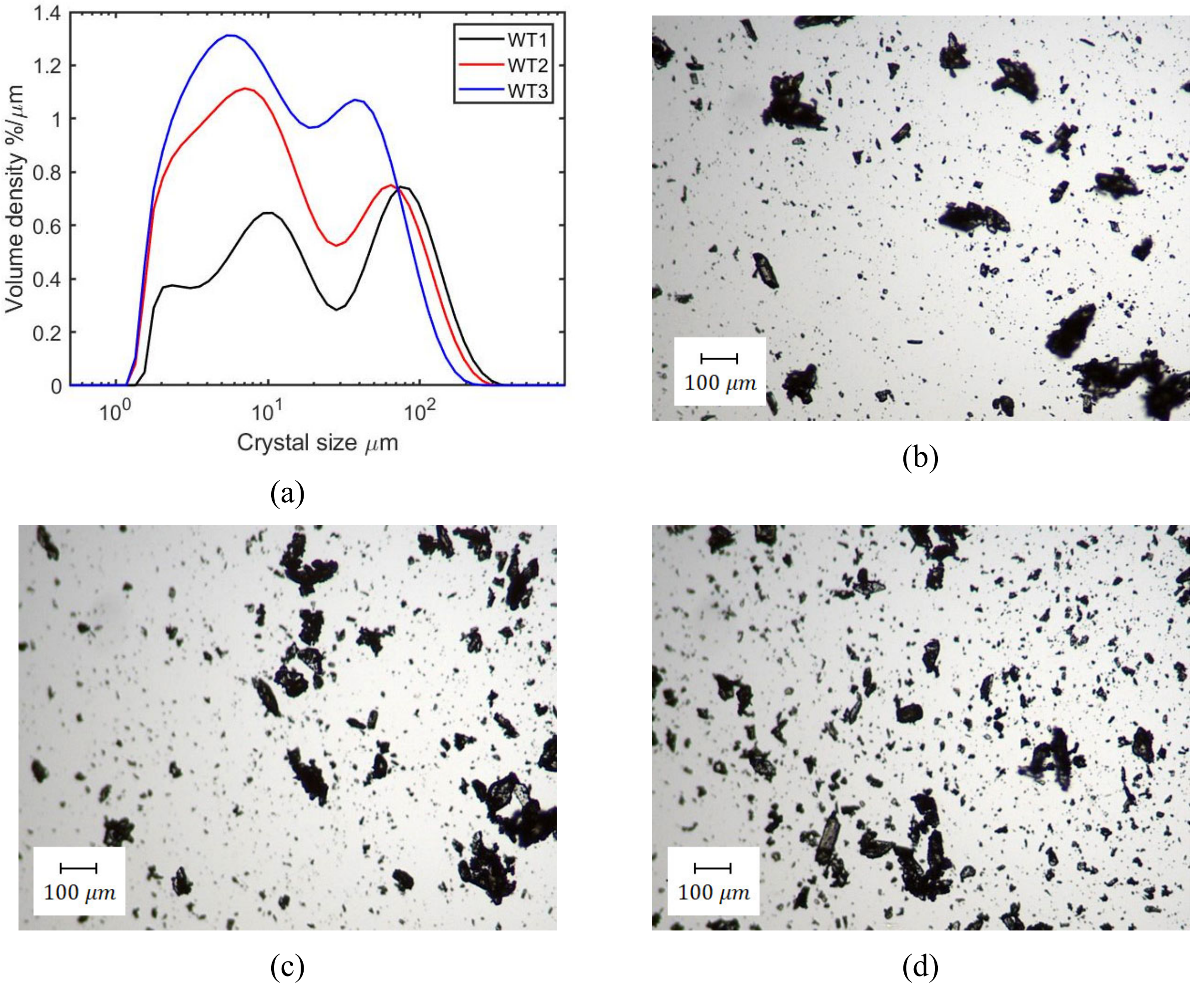

Figure 12. MasterSizer measurements (a) and product pictures of WT1 (b), WT2 (c) and WT3 d).

The offline CSD measurements are consistent with the FBRM trend that larger crystals remained in WT1 due to the limited milling time. The crystal agglomeration is reduced after the milling, but a large number of fine particles are generated, which is undesirable.

Therefore, the coarse mode with a rotor speed of 10000 rpm was selected for the subsequent studies, which focused on using temperature cycling to remove the generated fines and reduce overall agglomeration.

8. Dynamic Vapour Sorption of Form A

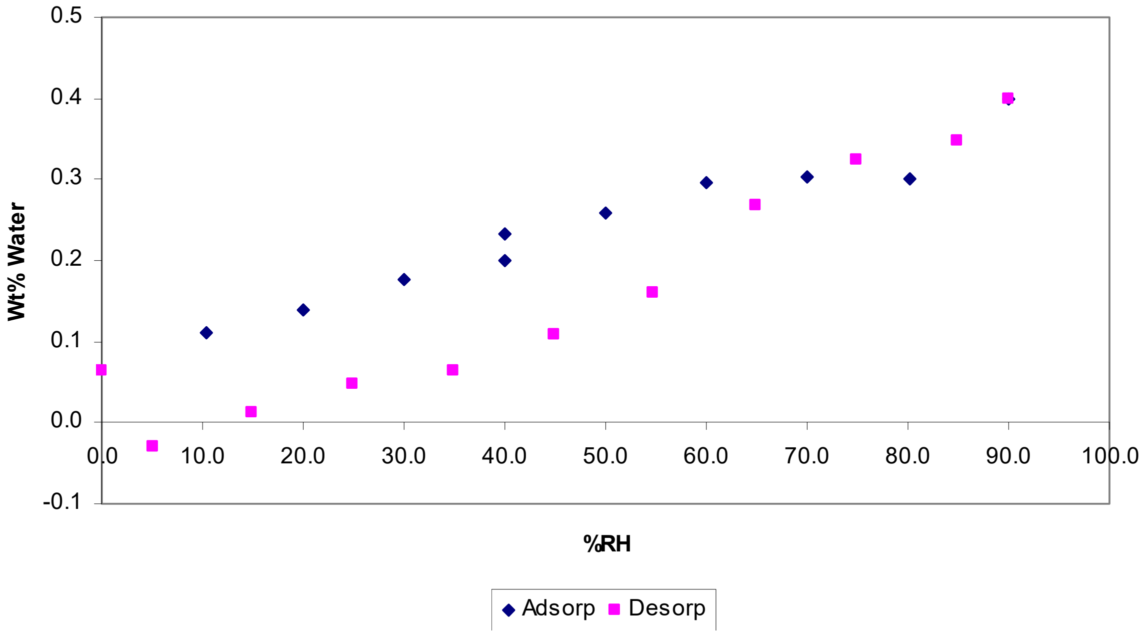

Figure 13. The gravimetric moisture sorption curve of Compound X starting material, Form A. The curve showed the form to be slightly hygroscopic adsorbing 0.4 wt% water at 90% RH.
